# A cryptic hydrophobic pocket in the polo-box domain of the polo-like kinase PLK1 regulates substrate recognition and mitotic chromosome segregation

**DOI:** 10.1038/s41598-019-50702-2

**Published:** 2019-11-04

**Authors:** Pooja Sharma, Robert Mahen, Maxim Rossmann, Jamie E. Stokes, Bryn Hardwick, David J. Huggins, Amy Emery, Dominique L. Kunciw, Marko Hyvönen, David R. Spring, Grahame J. McKenzie, Ashok R. Venkitaraman

**Affiliations:** 1 The Medical Research Council Cancer Unit, University of Cambridge, Hills Road, Cambridge, CB2 0XZ United Kingdom; 2 Department of Biochemistry, University of Cambridge, 80 Tennis Court Road, Cambridge, CB2 1GA United Kingdom; 3 Department of Chemistry, University of Cambridge, Lensfield Road, Cambridge, CB2 1EW United Kingdom; 4 Cavendish Laboratory, University of Cambridge, Cambridge, CB3 0HE United Kingdom

**Keywords:** Mitosis, Kinetochores, Kinases, Phosphoproteins, Small molecules

## Abstract

The human polo-like kinase PLK1 coordinates mitotic chromosome segregation by phosphorylating multiple chromatin- and kinetochore-binding proteins. How PLK1 activity is directed to specific substrates via phosphopeptide recognition by its carboxyl-terminal polo-box domain (PBD) is poorly understood. Here, we combine molecular, structural and chemical biology to identify a determinant for PLK1 substrate recognition that is essential for proper chromosome segregation. We show that mutations ablating an evolutionarily conserved, Tyr-lined pocket in human PLK1 PBD trigger cellular anomalies in mitotic progression and timing. Tyr pocket mutations selectively impair PLK1 binding to the kinetochore phosphoprotein substrate PBIP1, but not to the centrosomal substrate NEDD1. Through a structure-guided approach, we develop a small-molecule inhibitor, Polotyrin, which occupies the Tyr pocket. Polotyrin recapitulates the mitotic defects caused by mutations in the Tyr pocket, further evidencing its essential function, and exemplifying a new approach for selective PLK1 inhibition. Thus, our findings support a model wherein substrate discrimination via the Tyr pocket in the human PLK1 PBD regulates mitotic chromosome segregation to preserve genome integrity.

## Introduction

The human polo-like kinase 1 (PLK1) is a key regulator of chromosome segregation during mitosis, through its essential biological functions in chromosome condensation^1^, cohesin dissociation from chromosomes^2^, mitotic entry^3^, centrosome maturation^4^, kinetochore function^5^, spindle assembly^6,7^, and exit from mitosis^8–10^. These disparate functions are mediated through the phosphorylation of proteins that bind to chromatin, centrosomes or kinetochores. How these multiple roles can be precisely choreographed during chromosome segregation remains unclear, but mounting evidence suggests that PLK1 substrate recognition plays a key role. Thus, human PLK1 adopts a structure^11^ that consists of an amino (N-)terminal kinase catalytic domain, with two repeats of the so-called ‘polo box’ motif juxtaposed to form a single functional unit called the Polo-Box Domain (PBD) positioned at the carboxyl (C-) terminus. A similar architecture is adopted by the related human polo-like kinases PLK2, PLK3 and PLK4, which also contain a structurally related PBD. The PBD recognizes phospho (p)Ser/Thr protein substrates for the kinase catalytic activity of PLKs^12^. Such PBD substrates are typically primed by a prior phosphorylation event catalyzed by other kinases such as CDK1^13^, or in some cases, by PLK1 itself^14,15^. Several lines of evidence indicate that phosphoprotein substrate recognition by the PBD is coordinated with substrate phosphorylation by the kinase catalytic domain to determine the spatio-temporal dynamics of PLK1 localisation and function during chromosome segregation^12,16–18^.

The crystal structure of a substrate peptide in complex with the human PLK1 PBD^12^ reveals that pSer/Thr residues in the phosphopeptide substrate bind to a groove between the two polo boxes, with the phosphate moiety making contacts with two crucial residues, His 538 and Lys 540 in the PBD. Mutations in the PBD that preclude these key contacts (H538A and K540M) disrupt phosphopeptide substrate engagement and the cellular functions of PLK1^19^. How substrate residues other than pSer/pThr dictate substrate discrimination to choreograph PLK1 function during chromosome segregation remains an important unresolved issue, however.

Intriguingly, recent crystallographic studies have identified a hydrophobic pocket within the human PLK1 PBD, located adjacent to the phosphosubstrate binding groove, which may regulate substrate recognition. The pocket is evident only in certain structural conformers, suggesting that it may adopt an open conformation in which it could accommodate residues from PLK1 substrates, or alternatively, a closed conformation precluding such interactions^20^. Seven hydrophobic amino acid residues line the pocket; whilst V415, L478, and F482 cover the bottom of the pocket, the Tyr residues Y417, Y421, Y481 and Y485, form its sides^20^. We find that these Tyr residues are conserved across eukaryotic members of the polo-like kinase family, prompting us to term this structural feature the “Tyr pocket”. The proximity of the Tyr pocket to the phosphosubstrate binding groove, and its ability to adopt an open conformation, suggest that it may assist in the recognition of PBD substrates that contain hydrophobic side chains, enabling substrate discrimination. For instance, the Tyr pocket is involved in the binding of a peptide derived from the PLK1 substrate, PBIP1^20^, which contains an Phe71 residue that engages the Tyr pocket, as well as with peptidic ligands or chemical fragments that bind to the PBD^21–25^. However, the functional significance of PLK1 substrate recognition via the Tyr pocket during mitotic chromosome segregation remains uncertain.

We have therefore combined molecular, structural and chemical biology to address this question. Here, we report that the Tyr pocket of the PLK1 PBD exerts an essential function in regulating the timing of progression through mitosis. We distinguish a PLK1 substrate that engages the Tyr pocket (PBIP1) from another that does not (NEDD1). We also identify a small-molecule chemical ligand for the Tyr pocket that recapitulates chromosome segregation defects induced by genetic mutations that ablate the pocket. Collectively, our results suggest a model in which the Tyr pocket engages non-phosphorylated residues in phosphoprotein substrates to choreograph PLK1 activity during chromosome segregation. Our results also provide a structural and functional rationale for targeting the Tyr pocket to create selective chemical inhibitors that modulate substrate recognition by the polo-like kinases.

## Results

### Mutations ablating the Tyr pocket of the PLK1 PBD inhibit viability

**Figure 1:**
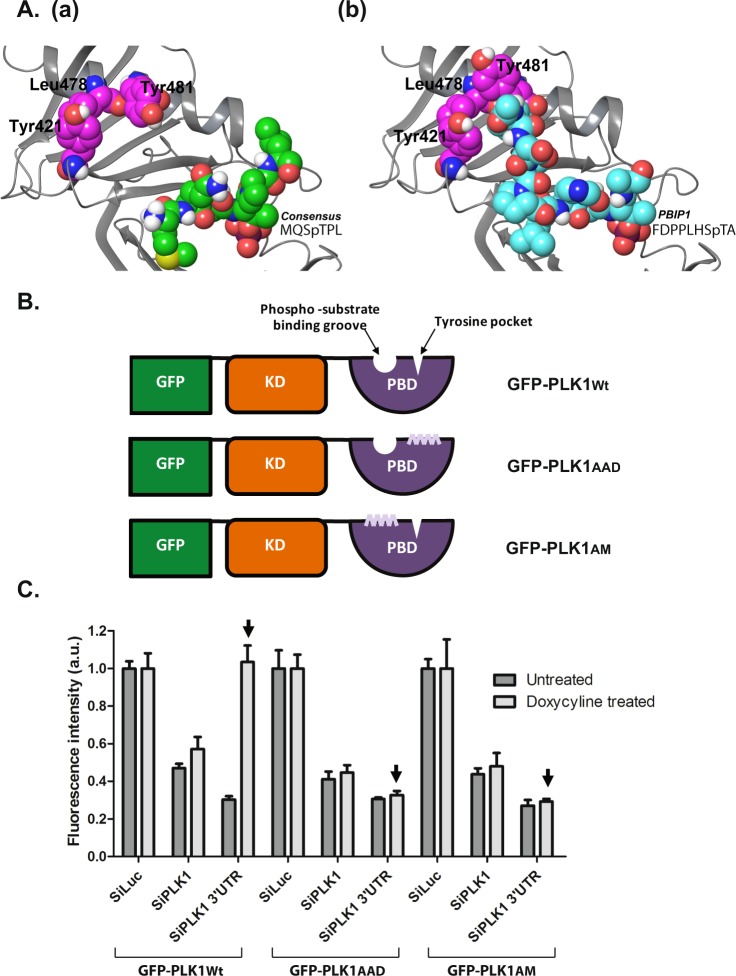
Mutations ablating the Tyr pocket of the PLK1 PBD inhibit cell viability. (**A**) The polo-box domain of PLK1 in complex with (a) the consensus sequence phosphopeptide MQSpTPL from PDBID 1Q4K and (b) the PBIP1 phosphopeptide FDPPLHSpTA from PDBID 3P37. The protein is represented as grey ribbons. The two phosphopeptides are displayed in space-filling with green and cyan heavy atoms respectively. The tyrosine pocket residues Tyr421, Leu478, and Tyr481 are labelled and displayed in space-filling with magenta heavy atoms. Only polar hydrogens are shown. (**B**) A schematic showing GFP -tagged PLK1_Wt/AAD/AM_ used in the study. KD = Kinase Domain, PBD = Polo-Box Domain. (**C**) The graph shows cell viability of GFP-PLK1_Wt/AAD/AM_ (as indicated) at the end of 48 h after transfection of siRNA and induction with doxycycline measured using CellTiter-Blue® reagent. The overexpression of GFP-PLK1_Wt_ but not GFP-PLK1_AAD_ or GFP-PLK1_AM_ is able to completely overcome the viability defect caused by the knockdown of endogenous PLK1 using siPLK1 3′UTR (see arrowheads). As expected, silencing of both overexpressed GFP-PLK1Wt/AAD/AM as well as the endogenous PLK1 has deleterious effects on cell viability. Each bar is a mean of 3 replicates ± S.D.

We first devised an experimental system in which to address the functional significance of the Tyr pocket of the PLK1 PBD. Structural modelling of the Tyr pocket defined three point mutations - Y421 to A, L478 to A and Y481 to D – predicted to ablate the Tyr pocket whilst preserving the structural integrity of the nearby phospho-substrate binding groove (Fig. 1A and Methods). Accordingly, we used site-directed mutagenesis to create full-length human PLK1 harbouring these three mutations (GFP-PLK1_AAD_), and (N)-terminally fused to enhanced green fluorescent protein (GFP). For comparison, we also created plasmids encoding similar GFP-tagged PLK1 constructs encoding the wild-type protein (GFP-PLK1_Wt_), or a well-characterized PLK1 mutant protein harbouring mutations (H538 to A, and K540 to M) affecting residues critical for the phospho-substrate binding groove (GFP-PLK1_AM_) (Fig. 1B). Three HeLa cell lines inducibly expressing either GFP-PLK1_Wt_, GFP-PLK1_AM_ or GFP-PLK1_AAD_ upon exposure to doxycycline (Dox) were created using single integration into a defined genomic locus (with the Flp-In^TM^ T-REx^TM^ system). These cells inducibly express wild-type or mutant PLK1 proteins as detected by immunoblotting or fluorescence microscopy (Supplementary Fig. S1).

Next, we depleted endogenous PLK1 expression in these cell lines using short-interfering (si)RNAs directed against the 3′ untranslated region (UTR) of the PLK1 transcript (Table S2), which is absent from the constructs encoding GFP-PLK1_Wt_, GFP-PLK1_AM_ or GFP-PLK1_AAD_. Indeed, in cells treated with siRNA against the PLK1 3′ UTR, and induced to express GFP-PLK1 by exposure to a pulse of 0.1 mg.ml^−1^ Dox, we confirmed that GFP-PLK1_Wt_, GFP-PLK1_AAD_ or GFP-PLK1_AM_ proteins were expressed between 24–48 h of induction, although endogenous PLK1 was no longer detectable (Supplementary Fig. S2). As a control, we confirmed that both endogenous and GFP-PLK1 expression can be depleted using siRNAs (Table S2) directed against a region of the PLK1 transcript present in both the endogenous and induced species (Supplementary Fig. S2).

PLK1 is essential for progression through the cell cycle, and it is well established that siRNA-mediated depletion of endogenous PLK1 suppresses cell division^26–28^. We therefore tested whether Dox-induced expression of wild-type or mutant GFP-PLK1 proteins could complement defects in cell proliferation triggered by depletion of endogenous PLK1. As expected, GFP-PLK1_Wt_ could complement suppressed cell proliferation, whereas the GFP-PLK1_AM_ mutant, in which the phospho-substrate binding groove has been ablated, did not. Interestingly, the GFP-PLK1_AAD_ mutant protein, in which the Tyr pocket has been ablated, also did not support cell viability (Fig. 1C). These observations suggest that the Tyr pocket of the PLK1 PBD is essential for progression through the cell cycle.

### Mutations ablating the Tyr pocket of the PLK1 PBD perturb the dynamics of its intracellular localisation during the cell cycle

**Figure 2:**
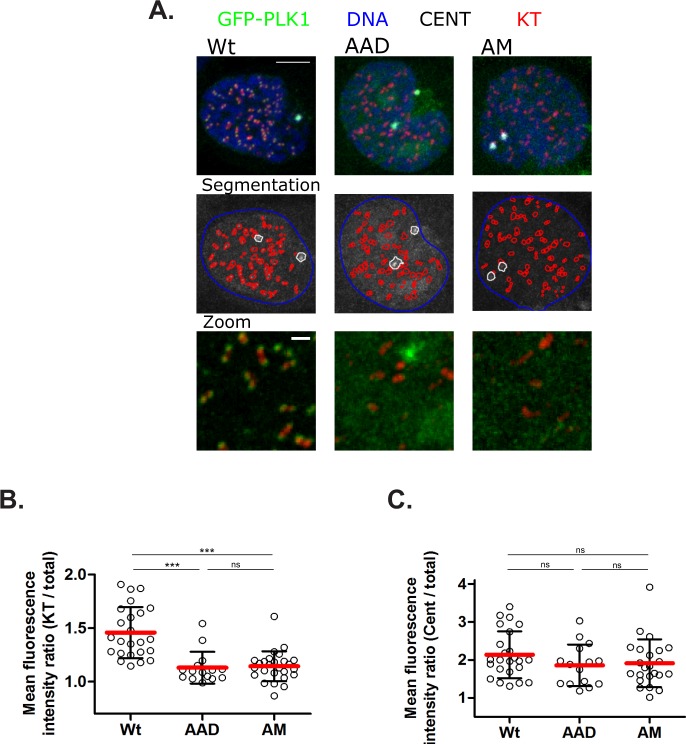
Mutations ablating the Tyr pocket of the PLK1 PBD perturb the dynamics of its intracellular localisation during the cell cycle. (**A**) Representative maximal-intensity projections of kinetochores (KT) in red, centrosomes (CENT) in white, GFP-PLK1_Wt/AAD/AM_ in green and DNA in blue used for quantification of GFP-PLK1 intensity in (**B,C**). Cells were treated with Dox (0.5 mg.ml^−1^) for 7 h, fixed and stained for kinetochores with CREST antiserum, centrosomes with anti-Pericentrin and DNA with Hoechst 33342 and analysed by immunofluorescence microscopy for GFP signal in prophase mitotic cells. Segmentation images show computationally generated segmentations of the DNA (blue), KTs (red) and CENTs (white) used for analysis. Scale bar is 5μm on top panel and 1μm in bottom panel. The images were smoothed, and brightness and contrast settings are constant throughout. (**B**) Quantification of intensity ratios of GFP-PLK1_Wt/AAD/AM_ on CREST-stained kinetochores (KT) normalized to the corresponding GFP-PLK1 expression in cells. Data from each cell is represented as a hollow circle, horizontal line (red) indicates mean intensity ratio and error bars indicate ± S.D. Statistical analysis was done using non-parametric, Mann-Whitney two-tailed test with 95% confidence interval, ***p < 0.0001, ns = not significant. (**C**) Quantification of intensity ratios of GFP-PLK1_Wt/AAD/AM_ on Pericentrin-stained Centrosomes (CENT) normalized to the corresponding GFP-PLK1 expression in cells. Data from each cell is represented as a hollow circle, horizontal line (red) indicates mean intensity ratio and error bars indicate ± S.D. Statistical analysis was done using non-parametric, Mann-Whitney two-tailed test with 95% confidence interval, ns = not significant.

PLK1 exhibits a distinctive pattern of localisation and redistribution to different sub-cellular structures during the cell cycle, decorating centrosomes^29,30^, kinetochores^13,31–33^, spindle mid-zone fibers^34,35^, and the cytokinetic midbody^34,36^, during distinct cell cycle phases. Mutations vitiating the phospho-substrate binding groove of the PLK1 PBD are reported to perturb the dynamics of PLK1 localisation^17,37^, suggesting that substrate recognition via the PBD is responsible for its intracellular localisation. Consistent with this notion, the localisation of GFP-PLK1_Wt_ to kinetochores (Fig. 2A,B) and centrosomes (Fig. 2A–C) mirrors that reported for endogenous PLK1 protein^30^. In contrast, GFP-PLK1_AM_ is decreased at kinetochores (Fig. 2A,B) as previously reported^38^, but persists at centrosomes (Fig. 2A–C)^18,37^, distinguishing the contribution of substrate recognition by the PBD to these mitotic structures. Similarly, GFP-PLK1_AAD_ exhibits reduced accumulation on CREST-stained kinetochores (Fig. 2A,B), although its localisation to centrosomes (Fig. 2A–C and Supplementary Fig. S3) remains unimpaired. PLK1 is recruited to centrosomes via the interaction of its PBD to multiple phosphoproteins^39–41^. Thus, our finding that both GFP-PLK1_AM_ and GFP-PLK1_AAD_ retain their centrosomal localisation suggests that these mutations do not grossly perturb the folding of the mutant PBDs, or their ability to bind at least a subset of substrates. Remarkably however, both mutants decrease localisation to kinetochores, which again involves PBD interaction with different phosphoprotein partners^5,33,42,43^, suggesting that the mutations selectively perturb PBD interactions with other substrates.

### Mutations ablating the Tyr pocket of the PLK1 PBD impair mitotic progression

PLK1 depletion using siRNA, or inhibition of its enzymatic activity using chemical inhibitors, triggers mitotic arrest and defects in chromosome segregation^8,26,44,45^. We therefore tested the contribution of the Tyr pocket to these functions. As expected, depletion of endogenous PLK1 protein induces mitotic arrest, marked by a sharp increase (range, 31.7–34.7%) in the percentage of cells stained for the mitotic marker, phospho-histone H3 (pH3). Induced expression of GFP-PLK1_Wt_ overcomes mitotic arrest, reducing the percentage of pH3-stained mitotic cells to 9 ± 0.8%. However, neither the GFP-PLK1_AM_ nor GFP-PLK1_AAD_ mutant could overcome mitotic arrest induced by depletion of endogenous PLK1, indicating that the Tyr pocket as well as the phospho-substrate binding groove of the PBD are essential for this function (Supplementary Fig. S4).

**Figure 3:**
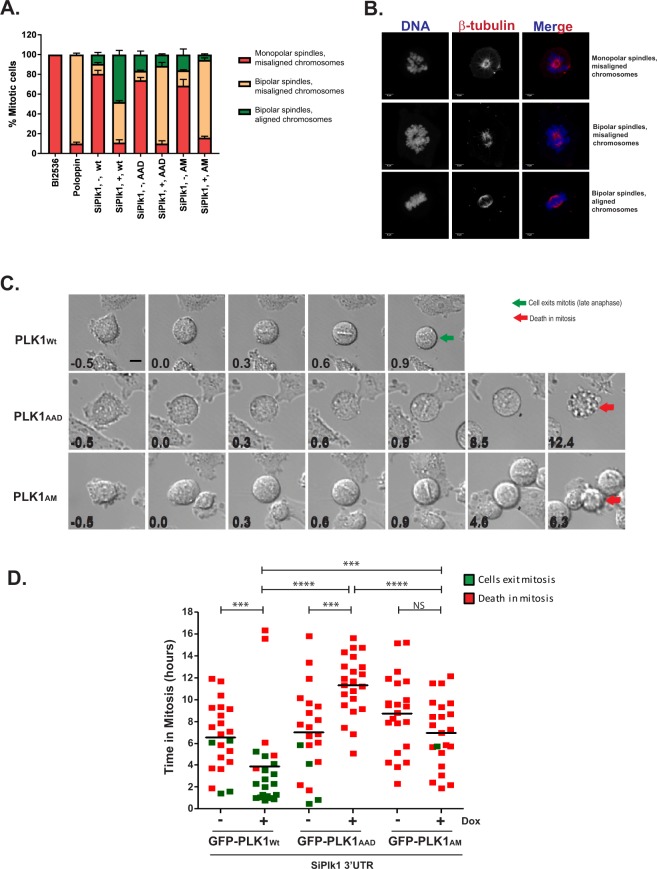
Mutations ablating the Tyr pocket of the PLK1 PBD impair mitotic progression. (**A**) HeLa cell lines inducibly expressing GFP-PLK1_Wt/AAD/AM_ were treated with siPlk1 3′UTR both with ( + ) and without (-) Dox or BI2536 or Poloppin as indicated and fixed 24 h later. The fixed cells were stained for spindle microtubules (β-tubulin) and DNA and analysed by microscopy. Mitotic cells were identified by spindle organisation and chromatin condensation, and scored under three categories *viz*., (1) monopolar spindles and misaligned chromosomes; (2) bipolar spindles with misaligned chromosomes; and (3) bipolar spindles with aligned chromosomes. Data is from two independent experiments, totalling at least 200 cells per condition. Quantification of cellular phenotype in each category in the histogram is represented as mean ± S.D. Treatments with BI2536 and Poloppin were included as control for categories (1) and (2) respectively. (**B**) Representative images of each category (as in **A**) used for scoring cells are shown. Scale bar 5μm. (**C**) Live cell time-lapse images of GFP-PLK1_Wt/AAD/AM_ expressing cells transfected with SiPlk1 3′UTR. Time in hours (h) before (−0.5 h) and after Nuclear Envelope Breakdown (NEBD) (t = 0.0 h) to late anaphase (green arrow)/ death (red arrow) is shown for each cell. Scale bar, 5μm. The images presented are representative of two independent experiments. (**D**) Duration in mitosis (h) from NEBD to late anaphase/ death, in single cell GFP-PLK1_Wt/AAD/AM_ cells by live cell imaging. Each filled-in square represents data from a single cell and those which complete mitosis are shown in green while those which undergo death are in red. The horizontal line indicates mean time (h) spent in mitosis before cell death. Statistical analysis was with Mann-Whitney two-tailed t-test; ns, not significant; ***p = 0.0003 to 0.0007; ****p < 0.0001. The total number of cells followed in each condition is 22. The data presented is representative of two independent experiments.

More subtle distinctions between the contributions of PLK1 kinase activity versus the phospho-substrate binding groove and the Tyr pocket of the PBD domain are suggested by overexpression studies. Previous work has shown that small molecule inhibition of PLK1 kinase activity results in mitotic arrest with monopolar spindles, whereas inhibition of the PBD domain causes bipolar spindles with misaligned chromosomes^38,45,46^. Thus, depletion of endogenous PLK1 protein causes monopolar spindle arrest with defects in chromosome congression (74.3 ± 6.4%) (Fig. 3A,B). Induced expression of either GFP-PLK1_Wt,_ or the PBD mutants GFP-PLK1_AAD_ or GFP-PLK1_AM_, counteracted these defects in differing ways. Induced expression of GFP-PLK1_Wt_ counteracts defects in both chromosome congression and bipolar spindle formation, with 48 ± 4.3% cells showing normal chromosomal congression with bipolar spindles (Fig. 3A). However, while GFP-PLK1_AAD_ or GFP-PLK1_AM_ overexpression restores bipolar spindle formation (respectively in 78.5 ± 2.5% or 78.5 ± 1.5% of cells), it is less effective in counteracting chromosome congression defects, with only 11.5 ± 0.7% or 5.5 ± 0.7% of cells respectively showing normal chromosomal congression (Fig. 3A). These results suggest that the Tyr pocket and the phospho-substrate binding groove of the PBD are both essential for normal chromosome congression but not bipolar spindle formation, whereas PLK1 kinase activity is essential for both processes.

We therefore analysed PLK1-depleted cells overexpressing either GFP-PLK1_AAD_ or GFP-PLK1_AM_ by single-cell imaging to discern mitotic progression. As expected, PLK1-depleted cells entered mitosis but did not complete division and instead, undergo prolonged arrest (6.5–9.13 h) before cell death (Fig. 3C,D). Induced expression of GFP-PLK1_Wt_ but not GFP-PLK1_AAD_ or GFP-PLK1_AM_ mutants overcomes the defect, permitting normal mitotic exit (Fig. 3C,D and Supplementary videos M1–3), confirming that the PLK1 PBD is essential for this process.

During mitosis, cells overexpressing GFP-PLK1_AAD_ or GFP-PLK1_AM_ mutants maintain a prometaphase arrest accompanied by significant chromosomal movement and failure to align chromosomes on the metaphase plate (Fig. 3C**)**. However, cells overexpressing GFP-PLK1_AAD_ persist longer in mitosis (11.5 h) before cell death compared to cells overexpressing GFP-PLK1_AM_ (7.1 h) (Fig. 3D). Collectively, these observations raise the possibility that the phospho-substrate binding groove and Tyr pocket make distinct, but essential, contributions during chromosome segregation.

### Differential engagement of PLK1 PBD substrates via the Tyr pocket

**Figure 4:**
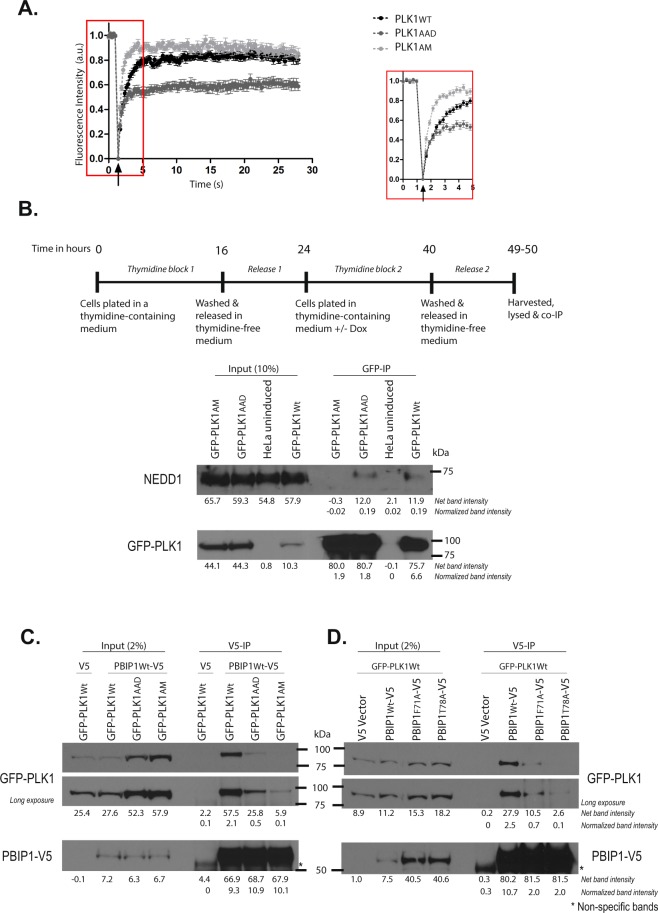
Differential engagement of PLK1 PBD substrates via the Tyr pocket. (**A**) FRAP analysis of interphase centrosomes in live cells. GFP-PLK1_Wt/AAD/AM_ at the centrosome was photobleached and its recovery monitored at 0.2 s intervals. The median t_1/2_ time taken for 50% maximal recovery for GFP-PLK1_Wt/AAD/AM_ were found to be 0.7 s, 0.4 s and 0.3 s respectively. The arrowhead indicates the point of photobleaching. An inset shows data points for the first 5 seconds including photobleaching and recovery time of GFP signal. (**B**) HeLa cells expressing GFP-PLK1_Wt/AAD/AM_ were synchronized in mitosis by double thymidine block and released as shown in the experimental schedule. The cell lysates were immunoprecipitated using GFP-Trap® beads to pull down GFP-PLK1_Wt/AAD/AM_ and analysed by immunoblotting. The data presented is representative of two independent experiments. (**C**) HeLa cells expressing GFP-PLK1_Wt/AAD/AM_ were transfected with PBIP1_Wt_-V5 or pcDNA™3.1/V5-HisA vector (or V5 vector) and harvested 24 h later. PBIP1_Wt_-V5/ V5 was pulled down from the lysates using V5 antibody and immunoprecipitates were analysed by immunoblotting. Asterisks (*) indicate cross-reacting bands. The data presented is representative of two independent experiments. (**D**) HeLa GFP-PLK1_Wt_ overexpressing cells were transfected with either PBIP1_Wt/F71A/T78A_-V5 or V5 vector and harvested 24 h later. PBIP1_Wt/F71A/T78A_-V5 was pulled down from the lysates using V5 antibody and analysed by immunoblotting. Asterisks (*) indicate cross-reacting bands. The data presented is representative of two independent experiments.

Dynamic analysis of GFP-PLK1 recruitment to mitotic structures using fluorescence recovery after photobleaching (FRAP) reveals differences in the function of the phospho-substrate binding groove and the Tyr pocket of the PBD. FRAP measures the speed and extent of the recovery of fluorescence at specific regions within living cells, after photobleaching of these regions with an intense but brief pulse of laser light (Supplementary Fig. S5). As previously reported^37^, differences in the speed of fluorescence recovery from GFP-PLK1_Wt_ and GFP-PLK1_AM_ (Fig. 4A, median time to 50% recovery (t_1/2_) 0.7 s (GFP-PLK1_Wt_) or 0.3 s (GFP- PLK1_AM_) are consistent with the faster diffusion of the GFP-PLK1_AM_ mutant protein, which cannot bind tightly to PBD substrates. In other words, the slow fluorescence recovery exhibited by GFP-PLK1_Wt_ indicates that its diffusion is limited by binding to other proteins such as PBD substrates, whereas the rapid fluorescence recovery of the GFP-PLK1_AM_ mutant is consistent with the loss of binding to PBD substrates^37^. Notably, however, fluorescence recovery by the GFP-PLK1_AAD_ mutant occurred with kinetics slower than GFP-PLK1_AM_ (medians t1/2 0.4 s vs t1/2 0.3 s respectively; p = 0.015, Mann-Whitney test) but more rapid than GFP-PLK1_Wt_ (median t1/2 0.4 s vs 0.7 s; p = 0.013; Mann-Whitney test). This observation suggests that mutations ablating the Tyr pocket may affect PLK1 binding to some, but not all, PBD substrates.

We tested this possibility using a biochemical approach. The canonical PLK1 PBD substrate, NEDD1 (Neural precursor cell Expressed Developmentally Downregulated gene1), is a centrosomal protein primed by CDK1 phosphorylation^47^ to bind the PBD during mitotic progression^48^. Consistent with this interaction, we find that GFP-PLK1_Wt_ can be co-immunoprecipitated with NEDD1 in extracts prepared from cells synchronized in mitosis (Fig. 4B, final right-hand lane, and Supplementary Fig. S6). Anti-NEDD1 reproducibly detects a modest but clear signal in the coimmunoprecipitates (relative intensity 0.2, normalized to its respective input lane). In contrast, the GFP-PLK1_AM_ mutant fails to interact with NEDD1 under similar conditions (relative intensity 0). Interestingly, GFP-PLK1_AAD_ retains binding to NEDD1 (relative intensity 0.2), demonstrating that the PLK1-NEDD1 interaction during mitosis is mediated via the phospho-substrate binding groove - but not the Tyr pocket - of the PBD. This observation accords with FRAP data wherein GFP-PLK1_AM_ shows faster recovery than GFP-PLK1_AAD_ than GFP-PLK1_Wt_.

We have previously shown by X-ray crystallography that the Tyr pocket of the PBD may adopt an open conformation in which certain phosphopeptide substrates containing hydrophobic residues that contact the Tyr pocket are accommodated^20^. One such substrate is PBIP1 (Polo-Box Interacting Protein 1), in which a phosphopeptide motif surrounding pThr78 binds to the PLK1 PBD to mediate its recruitment to the kinetochores^33^. We have previously shown in a crystal structure of PLK1 PBD bound to the PBIP1 phosphopeptide Phe(71)-Asp-Pro-Pro-Leu-His-pThr(78)-Ala that the hydrophobic residue, Phe71, contacts the Tyr pocket in the open conformation^20^.

We therefore used a biochemical approach to test the contribution made by the Tyr pocket in the PLK1-PBIP1 interaction during mitosis. Owing to the lack of a suitable commercially available antibody against PBIP1, we generated a carboxyl (C)-terminal V5 epitope-tagged PBIP1_Wt_ construct (PBIP1_Wt_ -V5), and over-expressed this tagged protein in cells harbouring either GFP-PLK1_Wt,_ or the GFP-PLK1_AM_ or GFP-PLK1_AAD_ mutants. Whereas PBIP1-V5 co-immunoprecipitates with GFP-PLK1_Wt_ protein, it does not bind to the GFP-PLK1_AM_ phosphosite mutant (Fig. 4C). Similarly, the interaction of PBIP1_Wt_ -V5 with the GFP-PLK1_AAD_ mutant is greatly reduced (Fig. 4C**)**. Reciprocal co-immunoprecipitation using an anti-GFP antibody shows that neither mutant can be detected in complex with PBIP1 (Supplementary Fig. S7), again consistent with greatly reduced interaction under these conditions. Thus, our results demonstrate an essential role for the PBD Tyr pocket in mediating the PLK1-PBIP1 interaction during mitosis.

To test whether the Phe71 residue in PBIP1 engenders a critical contact with the PBD Tyr pocket, we tested whether a mutant form of PBIP1-V5 in which Phe71 was mutated to Ala (PBIP1-F71A-V5) could bind to GFP-PLK1 proteins in the cellular milieu. As a control, we used a mutant form of PBIP1-V5 in which the phosphorylated residue, pThr78, was substituted with the non-phosphorylable amino acid Ala (PBIP1-T78A-V5), vitiating its ability to bind PLK1 PBD^33^. Indeed, the interaction of both the PBIP1-F71A-V5 (relative intensity 0.7) and PBIP1-T78A-V5 (relative intensity 0.1) mutants with GFP-PLK1_Wt_ in the cell extracts was markedly reduced when compared with wildtype PBIP1-V5 (relative intensity 2.5) (Fig. 4D). These results provide further evidence that engagement of the PBD Tyr pocket by Phe71 is essential for the PBIP1-PLK1 interaction during mitosis.

### A small-molecule inhibitor targeting the Tyr pocket in PLK1 PBD recapitulates pocket mutations

**Figure 5:**
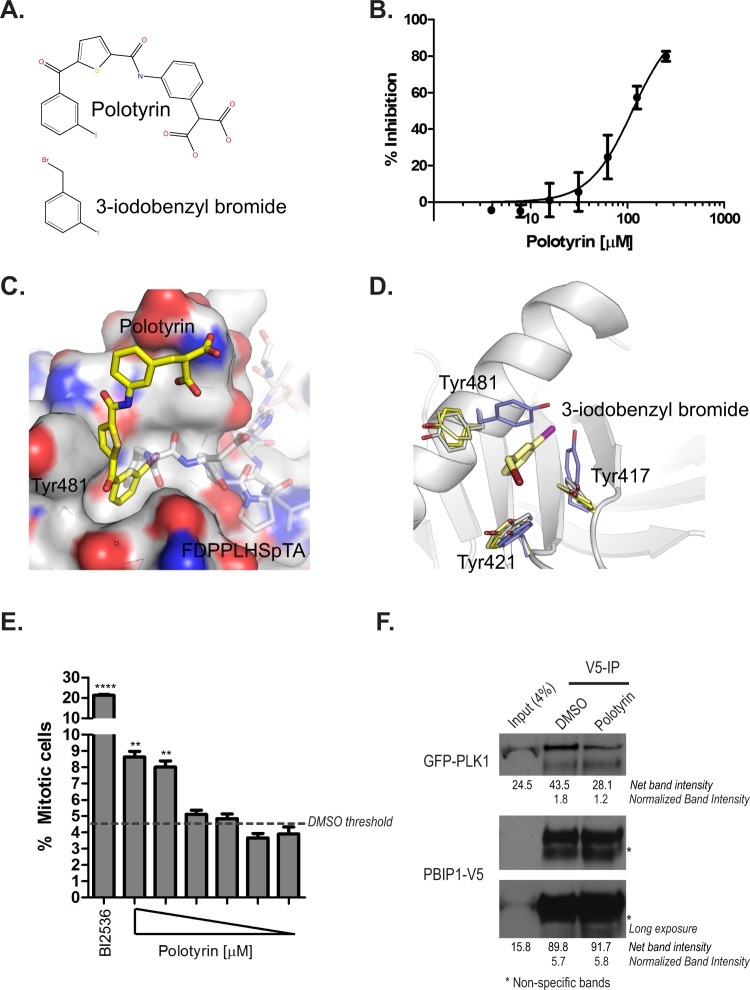
Targeting the Tyr pocket of the PLK1 PBD with a small-molecule inhibitor, Polotyrin. (**A**) The chemical structure of Polotyrin and 3-iodobenzyl bromide. (**B**) Polotyrin competitively inhibits the binding of a TAMRA- labelled-Glu-Thr-Phe(71)-Asp-Pro-Pro-Leu-His-pThr(78)-Ala-Ile-Tyr-Ala-Asp-Glu-acid phosphopeptide to the PLK1 PBD in Fluorescence Polarisation assay. (**C**) Crystal structure of PBD (surface rendering) with Polotyrin (stick model with yellow carbon atoms) shows how the compound binds in the Tyr-pocket that opens up on binding to long PBD substrates. Phosphopeptide FDPPLHSpTA from PBIP1 spanning the from Tyr pocket to the phospho-substrate binding groove is shown as transparent sticks for reference (PDB 3p37). (**D**) Complex of alpha-bromo-2-iodo-tolune (stick model with pale yellow carbons) bound to PBD with side chains of the aromatic residues aligning the Tyr pocket are shown as thin sticks (pale yellow). Tyr pocket lining residues from Polotyrin (yellow), FDPPLHSpTA (white) and LHSpTA (blue) complexes are also shown as well to illustrate structural changes in the pocket on binding to different ligands. (**E**) HeLa cells were treated with Polotyrin (at 1000, 800, 400, 200, 100 & 50 μM) and BI2536 (100 nM) for 24 h. Mitotic cells were scored as phospho-histone H3 positive cells and expressed as a percentage of the total number of Hoechst 33342-stained nuclei using a high-content screening platform as described earlier^55^. Each bar is a mean of three replicates ± S.D. and shows a dose-dependent increase in mitotic index upon treatment with Polotyrin; data from DMSO-treated cells is shown as a broken line. The data presented is representative of two independent experiments. Statistical analysis was done using Mann-Whitney two-tailed t-test; **p = 0.0029 to 0.0057; ****p < 0.0001. (**F**) HeLa GFP-PLK1_Wt_ cells were transfected with PBIP1_Wt_-V5 and harvested 24 h later. The lysates were incubated with Polotyrin (2 mM) or DMSO. PBIP1_Wt_-V5 was pulled down using V5 antibody and co-immunoprecipitates were analysed by immunoblotting.

Next, we used molecular modelling to design inhibitors that would block phosphopeptide recognition by the PBD. The chemical synthesis and characterization of one such molecule, Polotyrin, are described in Supplementary Methods. Polotyrin (Fig. 5A) inhibits the binding of a TAMRA-labelled PBIP1 phosphopeptide (Glu-Thr-Phe(71)-Asp-Pro-Pro-Leu-His-pThr(78)-Ala) to recombinant PLK1 PBD protein in a dose-dependent manner, with an IC50 of 115 ± 24 µM (Fig. 5B). To verify its binding pose, we soaked the molecule into PBD crystals and determined the structure of the complex. While designed to be a phospho-peptide mimetic, Polotyrin was found (Supplementary Figure S3) to occupy the Tyr pocket, with the iodophenyl moiety occupying the Tyr pocket and the thiophene ring stacking against Tyr481 (Fig. 5C). The binding opens the pocket, flipping both Tyr 481 and Tyr417 out of its way, akin to what a long PBD-binding phosphopeptide would do (Fig. 5D). The terminal group of Polotyrin, with an aromatic bi-carboxylic acid, makes limited interactions with the domain and is poorly resolved in the electron density, and does not engage with the phospho-Ser/Thr interaction site. Given this unexpected binding mode, we confirmed the binding of Polotyrin by soaking the crystals with an analogue of the Tyr-binding iodophenyl group, 3-iodobenzyl bromide. This fragment bound in the Tyr pocket in identical pose to Polotyrin (Fig. 5D), opening the pocket by shifting the two tyrosines. These findings confirm the binding site and binding mode for Polotyrin, as well as the Tyr pocket’s affinity for a iodophenyl group. The conformation of the Tyr pocket lining residues in Polotyrin and 3-iodobenzyl bromide complexes are virtually identical to those found in PBD in complex with the long FDPPLHSpTA peptide^20^. Thus, Polotyrin exemplifies a small-molecule inhibitor of phospho-substrate engagement with the PLK1 PBD via the Tyr pocket.

The cellular phenotypes elicited by Polotyrin exposure are consistent with its proposed mechanism of action. Polotyrin causes a dose-dependent increase in mitotic arrest in HeLa cells (Fig. 5E), marked by an increase in staining for the marker phospho-histone H3. Mitotic arrest is accompanied by characteristic anomalies in chromosome congression (Supplementary Fig. S8). Finally, Polotyrin treatment reproducibly but only slightly decreases the co-immunoprecipitation of PBIP1_Wt_-V5 with GFP-PLK1_Wt_ in cell extracts (Fig. 5F), consistent with the effects of mutation of F71A in PBIP1 (Fig. 4D). Thus, Polotyrin exhibits biological effects supporting its mechanism of action in the PLK1 Tyr pocket, albeit at relatively high doses consistent with its modest *in vitro* potency (IC50 ~115 µM), warranting further chemical optimization in future studies.

## Discussion

**Figure 6:**
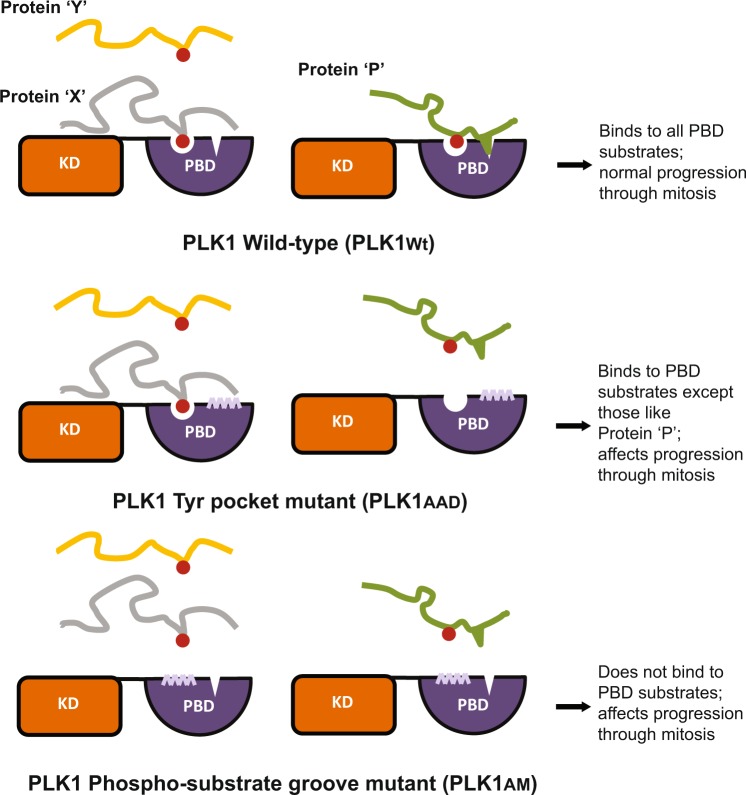
A model for the role of PLK1-Tyr pocket in differential substrate recognition and mitotic progression. The two classes of PBD phospho-substrates are shown as (1) those including proteins ‘X’ and ‘Y’ (e.g. NEDD1) and (2) others containing a hydrophobic amino acid residue proximal to the pS/pT residue, shown here as protein ‘P’ (e.g. PBIP1). PLK1_Wt_ binds to both categories of PBD-substrates; PLK1_AAD_ does not bind to protein ‘P’-like substrates while PLK1_AM_ binds none.

How the mitotic kinase PLK1 precisely recognizes and modifies multiple substrates to regulate sequential steps in chromosome segregation remains unclear. The findings we report here combine molecular, structural and chemical biology to define a previously unrecognized, novel function in chromosome segregation for a recently identified structural feature - the Tyr pocket – in the human PLK1 PBD. We provide a first line of evidence that the Tyr pocket plays an essential cellular role in the recognition of a class of PLK1 PBD substrates exemplified by PBIP1, distinct from those, like NEDD1, whose recognition depends solely on the previously characterized substrate binding groove (Fig. 6). Finally, we exploit this information to present evidence that small-molecule inhibitors targeting the Tyr pocket suffices to abrogate specific functions of PLK1 in dividing cells. Our findings have several important implications.

Our findings show for the first time that ablation of the Tyr pocket severely disrupts substrate recognition by the PLK1 PBD. Thus, the GFP-PLK1_AM_ or GFP-PLK1_AAD_ mutants exhibit defects in cell proliferation and mitotic progression, and in the localisation of PLK1 to kinetochores. These findings not only demonstrate that the Tyr pocket is essential for the cellular functions of PLK1, but also suggest that it does not play second fiddle to the well-characterized phosphosubstrate binding groove in substrate recognition.

Indeed, our findings strongly support the idea that a certain class of PLK1 PBD substrates, which may possess hydrophobic residues that engage the Tyr pocket adjacent to the key pSer/pThr, depend for their recognition on the integrity of this structural feature. Thus, PLK1_Wt_ binds to both the canonical substrates NEDD1 and PBIP1, whilst PLK1_AAD_ binds only to NEDD1, but PLK1_AM_ binds neither substrate (Fig. 4A–C). The potential functional significance of differential substrate recognition via the Tyr pocket is highlighted by several observations. Differences in the kinetics of fluorescence recovery after photobleaching exhibited by the GFP-PLK1_AAD_, GFP-PLK1_AM_ and GFP-PLK1_Wt_ proteins suggests that their capacity for substrate binding is in the order PLK1_Wt_ > PLK1_AAD_ > PLK1_AM_ (Fig. 4A), consistent with our biochemical experiments. Moreover, our observation that cells overexpressing GFP-PLK1_AAD_ persist for longer in mitosis before undergoing cell death when compared to those overexpressing GFP-PLK1_AM_ (Fig. 3D), as well as differences in mitotic progression between these settings, speak to the same conclusion, highlighting the importance of the Tyr pocket in the mitotic functions of human PLK1. Thus, our findings suggest a model in which the Tyr pocket acts in concert with the substrate binding groove to fine-tune the selective recognition of specific PLK1 substrates involved in mitotic progression.

A number of small-molecule inhibitors that disrupt protein-protein interactions of the PLK1 PBD with its cognate protein substrates have been developed^46,49–52^, although several of the earlier compounds are non-specific protein alkylators^39^. Furthermore, peptide-modified ligands have been developed which additionally span up to the Tyrosine pocket^25,51^. Here, we identify a novel small-molecule inhibitor, Polotyrin, which binds to the Tyr pocket (Fig. 5C). The cellular phenotypes elicited by Polotyrin are consistent with our conclusion from somatic cell genetics that targeting the Tyr pocket of PLK1 causes mitotic arrest (Fig. 5E), although further optimisation will be necessary to improve Polotyrin’s cellular activity. Thus, these findings provide proof-of-principle, and a structural blueprint, for the future design of a novel class of PLK1 inhibitors that modulate substrate recognition by the PLK1 PBD by targeting the Tyr pocket. Future studies using such chemical tools promise to enhance understanding of how PLK1 precisely coordinates the recognition and phosphorylation of multiple proteins that bind chromatin, centrosomes or kinetochores to regulate chromosome segregation during mitosis.

## Methods

### Cloning and transfection

GFP-Plk1_AAD_ was generated by sequential site-directed mutagenesis of GFP-Plk1_Wt_ (M. Daniels, Oxford University) using oligonucleotides in Supplementary Table S1. Plk1 from Plk1_Wt_, Plk1_AM_^25^ and Plk1_AAD_ were amplified using primers in Table S1 using AccuPrime Pfx DNA polymerase (ThermoFisher Scientific) following manufacturer’s instructions and cloned at XhoI and NotI restriction sites in pcDNA5/TO/FRT/GFP-Mps1 (kind gift from Professor Steve Taylor, University of Manchester) replacing Mps1 with Plk1.

Oligonucleotides (Supplementary Table S1) were used to amplify PBIP1_Wt_ from PBIP1 I.M.A.G.E. cDNA clone (Source Bioscience) using AccuPrime Pfx DNA polymerase as above. PBIP1_Wt_ was cloned in pcDNA™3.1/V5-HisA vector (ThermoFisher Scientific) at BamHI and XbaI restriction sites. This PBIP1_Wt_-V5 construct was then used to generate PBIP1_F71A_-V5 and PBIP1_T78A_-V5 by site-directed mutagenesis using oligonucleotides in Supplementary Table S1. All clones used in the study were verified by Sanger Sequencing (Source Bioscience). DNA transfection to generate stable cell lines was performed as described earlier^53^. Transfection of constructs (PBIP1_Wt/F71A/T78A_) was done using Lipofectamine 2000 (Invitrogen) according to manufacturers’ instructions. siRNA duplexes were purchased from Qiagen and MWG (Supplementary Table S2). SiRNA transfections were carried out at a concentration of 25 nM using DharmaFECT1 (Dharmacon, GE Healthcare) following manufacturer’s instructions.

### Molecular modelling of tyrosine pocket mutations

Structural analysis was performed on the PLK1 PBD complexed with the FDPPLHSpTA phosphopeptide from PBIP1 (PDBID 3P37). The protein structure was downloaded from the Protein Databank. Selenomethionines were changed to methionines and missing sidechains were added using Schrodinger’s Preparation Wizard, which was also used to check the orientations of the asparagine, glutamine, and histidine residues as well as the protonation state of all ionizable residues. All heteroatomic species such as buffer solvents and ions were removed. The hydrogen-atom positions were then built using Schrodinger’s Preparation Wizard and the force field parameters and partial charges were assigned from the OPLS force field^54^. Crystallographic water molecules were deleted.

Structural analysis was then performed to identify residues which could be mutated to abrogate phosphopeptide binding to the Tyrosine pocket but retain phosphopeptide binding to the phosphopeptide binding groove. Tyr417, Tyr421, Tyr481, and Tyr485 form the sides of the pocket. Val415 and Phe482 form the base of the pocket. Leu478 forms the back of the pocket. Val415, Tyr417, Phe482, and Tyr485 are relatively close to the phosphopeptide binding groove (all within 7 Angstroms of the backbone carbonyl of the −3 residue). Conversely, Tyr421, Leu478, and Tyr481 are approximately 10, 14, and 11 Angstroms from the backbone carbonyl of the −3 residue respectively (see Fig. 1A). These residues were selected for computational mutagenesis.

To avoid a combinatorial explosion, each of the three residues was mutated to only four other amino acids: alanine, serine, asparagine, and aspartate. These were selected for their small size. All 124 mutants were generated and the complexes with the FDPPLHSpTA phosphopeptide optimized using Schrodinger’s Embrace in each case. Predicted binding free energies between the PBD and the phosphopeptide were then estimated using Schrodinger’s MMGBSA approach^41^. Binding affinities were compared with the wild-type and the triple mutant Y421A/L478A/Y481D was selected as the most deleterious to FDPPLHSpTA phosphopeptide binding with a single change in charge.

### Cell culture, generation of stable cell lines and chemical treatments

HeLa FlpIn^TM^ T-REx^TM^ host cells (kind gift from Professor Steve Taylor, University of Manchester) were used for generating stable cell lines. The cells were grown at 37 °C under 5% CO_2_ in DMEM with GlutaMAX (Life Technologies), supplemented with 10% fetal calf serum, Zeocin^TM^ (Invitrogen) at 0.05 mg.ml^−1^ and Blasticidin (InvivoGen) at 4 μg.ml^−1^. Medium containing Hygromycin (InvivoGen) at 0.2 mg.ml^−1^ and Blasticidin were used for selection and maintenance of resistant clones which were pooled and expanded. For all experiments unless specified, doxycycline (Sigma-Aldrich) at 0.1 mg.ml^−1^ was used for induction of GFP-fusion protein.

For synchronisation, cells were arrested in early S-phase with Thymidine (Sigma, 2 mM) for 16 h, then released for 8 h in Thymidine-free medium, these were then arrested again with Thymidine for 16 h followed by release. As and where specified an overnight treatment with Nocodazole (Sigma) at 40 nM, BI2536 (Axon Medchem) at 100 nM and Poloppin^41,46^, at 100 µM was given.

### Immunoblotting and analysis

For immunoblotting, proteins were extracted in lysis buffer containing 50 mM HEPES (pH7.4), 100 mM NaCl, 0.5% NP-40, 10 mM EDTA, 20 mM β-glycerophosphate, 1 mM DTT, 1 mM sodium orthovanadate, 1 mM PMSF, protease inhibitor cocktail and phosphatase inhibitor (Roche). Extracts were resolved by SDS-PAGE, transferred to PVDF membrane. Ponceau S (Sigma-Aldrich) staining was done on the membrane prior to blocking to visualise uniform transfer of the samples. The membrane was blocked with TBS-Tween (50 mM Tris pH 7.6, 150 mM NaCl, 0.1% Tween-20) plus 5% non-fat dried milk, and probed with primary antibodies overnight at 4 °C. Following that, the membrane was washed in TBS-Tween and incubated in secondary antibodies for 1 h at room temperature. The membrane was washed in TBS-Tween and signal was developed using ECL or ECL prime reagent (GE Healthcare). The intensity of protein bands in pull down experiments was quantified using ImageJ. For this, a region of interest (ROI) was created around a protein band in a grayscale image, and band intensity was measured. For each blot, the same frame (ROI) was used to make measurements of protein bands across all the lanes including the background. The pixel intensities were inverted for each band (255 – measured band intensity) including background. Net signal intensity was obtained by subtracting inverted background intensity. Normalised band intensity (as seen in figures) was expressed by taking a ratio of net signal intensity of IP band over its input control.

### Cell viability assay

Cells were seeded in 96-well plates at a density of 4000 cells per well in 100 µl of medium without antibiotics, 16 h later treated with doxycycline and siRNA simultaneously and the medium was replaced the next day. CellTiter-Blue®reagent (Promega) was diluted (1:10) and added to the medium 48 h after transfection. The plates were incubated at 37 °C for 2 h in the dark in a humidified 5% CO_2_ incubator. Cell viability was determined by increase in fluorescence signal using a plate reader with filter sets 590 ± 20/540 ± 20 (PheraStar, BMG).

### Immunofluorescence and image analysis

Cells were grown and fixed on μ-slides (Ibidi, 80826) with 4% formaldehyde (Agar Scientific) for 10 min. Cells were permeabilised with 0.1% Triton-100 (ThermoFisher Scientific), 0.1% Tween-20 (NBS Biologicals) in 1x PBS (PBS-Triton-Tween) for 10 min and blocked with 1% BSA (ThermoFisher Scientific) in PBS-Triton-Tween for 30 min. Antibodies were diluted in the blocking solution and cells were incubated in the dark for 1 h at room temperature. The cells were washed thrice with the blocking solution, and incubated with Alexa Fluor^TM^ conjugated secondary antibodies (Life Technologies) in the dark for 30 min at room temperature. The cells were washed twice with the blocking solution, and stained for 2 minutes with Hoechst 33342 (1:4000 diluted in 1XPBS, Invitrogen) and washed twice with 1X PBS. The samples were stored at 4 °C in the dark before microscopy. For Fig. 3A,B, mitotic phenotypes were counted manually under the microscope, and representative images were taken on a LeicaSP5 confocal microscope using a 100×1.4 NA/oil objective. For sub-cellular localisation of GFP-PLK1_Wt/AAD/AM_ (Fig. 2), the images were captured using a Zeiss880 confocal microscope using a Plan-apochromat 100×/1.46 NA oil objective with z-stacks of confocal slices taken at 0.5 μm intervals. Pixel intensities were never saturated and laser exposure and detector settings were identical between samples across an experiment. To computationally measure GFP-PLK1 levels at centrosomes and kinetochores on a per cell basis, maximum intensity projections of four colour 3D data were analysed in Cell Profiler. Segmentation of DNA by Hoechst 33342 staining was used to create a mask within which anti-CREST foci denoting kinetochores were detected by thresholding. Centrosomes marked by anti-PCNT staining were simultaneously detected within segmented cytoplasmic or nuclear regions of the same cells. To correct for variability in GFP-PLK1 expression level, values were normalized to mean cellular levels in the cytoplasm and nucleus. Segmentation was manually checked for accuracy.

### Mitotic index (MI) assay

MI assay was either performed in high content screening format as described in^55^ or by manually counting the phospho-histoneH3 staining per 100 Hoechst stained nuclei under the microscope.

### Live cell imaging

For time-lapse analysis, cells were plated in a chambered glass-bottom plate (Lab-Tek Chambered coverglass, catalogue no. 155383), transfected with siRNA and imaged in a heated chamber (37 °C and 5% CO_2_) using a 40×/0.5 NA objective on Leica Live cell microscope. Images were recorded every 5 min for up to 40 h. Data from live cells was analysed using Leica Lite AF software, between 16–33 h of siRNA transfection and induction of GFP-PLK1_Wt/AAD/AM_. Time in mitosis was determined from nuclear envelope breakdown (NEBD) to late anaphase.

### FRAP and image analysis

FRAP was essentially as described in Mahen *et al*.^39^, with some modifications. Cells were imaged in L15 CO_2_ independent media (Gibco) at 37 °C, in μ-slides (see above) on a Zeiss 880 confocal microscope with a Plan-apochromat 63×/NA 1.4 oil objective. One confocal slice was imaged with a pixel size of 0.082 µm × 0.082 µm × 1 µm in the x-y-z dimension respectively, with each image taking 0.2 s to acquire. Bleaching took 0.4 s in a circle of area 3.19 µm^2^ using 100% power of a 488 nm argon laser. Regions of interest were defined in Zen Black software and analysis was performed with EasyFRAP^56^ making corrections for background fluorescence and image bleaching. t1/2 s were calculated by fully normalising the data and fitting with a single exponential term as described in^56^.

### Co-Immunoprecipitation (co-IP)

0.5 to 2.0 mg of lysates was used for co-IP. GFP-pull down was performed using 10 μl GFP-Trap® (Chromotek) as per manufacturer’s instructions. V5 antibody was pre-incubated with G-beads (Dynabeads, Invitrogen) as per manufacturer’s instructions. V5-beads were incubated with the lysates for 2 h at 4 °C on a rotator to pull down PBIP1_Wt/F71A/T78A_-V5. Incubation with Polotyrin (2 mM) and corresponding control DMSO was done at this stage. The immune-complexes were washed thrice with the lysis buffer and denatured using LDS loading buffer (Invitrogen) and heating at 70 °C prior to resolving on SDS-PAGE.

### Antibodies

The following antibodies were used for immunoblotting or immunoflourescence; PLK1 (Invitrogen 33-1700; 1:1000), CREST (Europa, 1:1000), Pericentrin (Abcam 4448; 1:500) β-tubulin (Sigma-Aldrich D66; 1:1000), GFP (Clontech JL-8 632381; 1:1000), V5 (Invitrogen R96025; 2ul for IP & 1:5000 for immunoblotting) PhosphoS10-histone H3 (Abcam 14955; 1:2500), Nedd1 (Abcam 57336; 1:1000), β-actin (Sigma-Aldrich A5441; 1:10000), HRP-conjugated Mouse/Rabbit/ L-chain specific (Jackson ImmunoResearch Laboratories, Inc., 1:10000); Alexa Fluor^TM^ mouse 568, 594, human 594 and rabbit 633 secondary antibodies used for immunofluorescence (ThermoFisher Scientific; 1:500).

### Polotyrin discovery and synthesis

The binding poses of both PLHSpT and LHSpTA were identified by Yun *et al.*^57^. These structures were used as the starting point for the design of small molecule Plk1 inhibitors we termed phosphopeptidomimetics as they were designed to mimic the structure of LHSpT.

The formulation of *de novo* phosphopeptidomimetics began with the modelling of the LHSpT atomic arrangement in three-dimensional space. Correct positioning of LHSpT electronic features and overall shape was enabled through this conceptualisation process. Small molecules were then assembled to replicate the same electronic and shape features of LHSpT. This technique was used to identify several structures, chemical intuition was used to produce compound libraries. These libraries were docked into the prepared crystal structure of LHSpTA using the Schrodinger small molecule discovery suite and subsequently analysed by docking score and visually inspecting the docking pose. Two rounds of *in silico* docking and analysis were required for the discovery of small molecules such as polotyrin that warranted synthetic effort. The synthesis of Polotyrin is described in detail in the Supplementary Methods section.

## Supplementary information

Video M1

Video M2

Video M3

Supplementary Information

## Data Availability

Crystallographic data reported in this paper have been deposited in the Protein Data Bank database with identifiers 5NEI and 5NMM. All data, materials and associated protocols are available without restriction.
