# Supplementary material for: A cryptic hydrophobic pocket in the polo-box domain of the polo-like kinase PLK1 regulates substrate recognition and mitotic chromosome segregation
Source: Sci Rep. 2019 Nov 4;9:15930. doi: 10.1038/s41598-019-50702-2 (PMC6828814; doi:10.1038/s41598-019-50702-2)

Supplementary Information

A cryptic hydrophobic pocket in the polo-box domain of the polo-like kinase PLK1 regulates substrate recognition and mitotic chromosome segregation

Pooja Sharma, Robert Mahen, Maxim Rossmann, Jamie E. Stokes, Bryn Hardwick, David J. Huggins, Amy Emery, Dominique L. Kunciw, Marko Hyvönen, David R. Spring, Grahame J. McKenzie and Ashok R. Venkitaraman

Supplementary Methods.

Fluorescence Polarisation assay

Final concentrations of assay components used in binding assays were as follows: TAMRA-labelled PBIP1 phosphopeptide, 5-TAMRA-Glu-Thr-Phe(71)-Asp-Pro-Pro-Leu-His-pThr(78)-Ala-Ile-Tyr-Ala-Asp-Glu-acid 10nM; PLK1 PBD (aa345-603) 42nM (1.25ng/µl). Assays were carried out in PBS (pH 7.4) plus 0.03% tween. DMSO controls were run alongside all experimental compounds and percentage inhibition normalised to these controls. Compounds were titrated 2-fold from a top concentration of 250µM giving a maximum final concentration of DMSO in the assay of 0.25%. The total assay volume per well was 45µl. Experiments were performed in NBS black 384-well microtiter plates (Corning). All assay components were incubated together at 22°C for 20 minutes prior to Fluorescence Polarisation (FP) being read using a BMG PheraStar plate reader with a 540/590/590nm FP module and unbound 10nm TAMRA-labelled peptide set to a FP value of 35mP.

PBD structure determination

PBD (residues 371-594) of human Plk1 was expressed and purified as described in Sledz et al [20]. The purified PBD domain was crystallised in 100-200 mM K/NA Tartrate, 10-20% PEG3350. Crystals were soaked overnight with Polotyrin or 3-iodobenzyl bromide in the presence of 10% DMSO and 10% PEG8000 as cryoprotectant and crystals cryocooled in liquid N_2. Diffraction data was collected at Diamond Light Source

beamlines i24 and i03, the data was processed with XDS [57]. Structures were solved by molecular replacement using unliganded PBD structure (PDB code 3P2W) as the search model. The structure was refined briefly before electron density evaluated for the presence of clear additional density for the soaked ligand. The resulting complex structures were refined using phenix.refine [58], with manual rebuilding and validation in Coot [59]. The refined coordinates have been submitted to Protein Data Bank under accession codes 5NEI (complex with Polotyrin) and 5NMM (complex with 3-iodobenzyl bromide).

Synthesis and characterisation of Polotyrin: General information

All non-aqueous reactions were performed at room temperature under a constant stream of dry nitrogen using glassware that had been oven-dried overnight unless otherwise stated.

Room temperature (RT) refers to ambient temperature. All temperatures below 0 °C were that of the external bath. Temperatures of 0 °C were produced and maintained with an ice-water bath. Temperatures below 0 °C were produced and maintained using an acetone-dry ice bath.

All reagents and solvents were used as received unless otherwise stated. Where appropriate, reagents and solvents were purified using standard experimental techniques. Ethyl acetate and methanol were distilled under nitrogen with calcium hydride. Tetrahydrofuran was dried over Na wire and distilled, while under nitrogen, from a combination of calcium hydride and lithium aluminium hydride with triphenylmethane as indicator. Pet ether refers to the fraction of light petroleum ether that had a boiling point between 40 and 60 °C. Brine refers to a sat. aqueous NaCl solution.

Yields refer to spectroscopically and chromatographically pure compounds unless otherwise stated in the experimental text. Reactions were monitored using thin layer

chromatography performed on commercially prepared glass plates pre-coated with Merck silica gel 60 F_{254} and visualised by quenching of UV fluorescence (ν_{max} = 254 nm), iodine, potassium permanganate, *p*-anisaldehyde, vanillin, phosphomolybdic acid, ninhydrin or by liquid chromatography mass spectrometry (LCMS) using a Waters Micromass ZQ spectrometer. Retention factors (R_f) are quoted to 0.01. R_f values were not determined for carboxylic acids due to their propensity to stick to the baseline.

Column chromatography was carried out using Merck 9385 Keiselgel 60 SiO_2 (230-400 mesh) under a positive pressure of compressed air.

Lyophilisation was achieved by suspending the required residue in a MeCN-H_2O (1:1) solution which was cooled to -196 °C with liquid nitrogen. The frozen sample was concentrated using a Scanvac CoolSafe 100-9 Pro freeze dryer overnight.

Infrared spectra were recorded neat on a Perkin-Elmer 1600 FT IR spectrometer. Only absorption maxima (ν_{max}) of interest are reported in wavenumbers (cm^{-1}) with the following abbreviations: w, weak; m, medium; s, strong; br, broad.

Melting points were obtained on a Büchi B-545 melting point apparatus and are uncorrected.

Proton magnetic resonance spectra were recorded using an internal deuterium lock at ambient probe temperatures on the following instruments: Bruker Avance 400 CRYO QNP (400 MHz), Bruker Avance 400 QNP (400 MHz), Bruker Avance 500 CRYO (500 MHz). Chemical shifts (δ_H) are quoted in parts per million (ppm) to the nearest 0.01 ppm downfield of trimethylsilane (δ_H = 0) and are referenced to the residual non-deuterated solvent peak as follows: $CDCl_3$, 7.26 ppm; d_6-DMSO, 2.50 ppm. Integration, chemical shift, multiplicity (s, singlet; d, doublet; t, triplet; q, quartet; quint, quintet; m, multiplet; br, broad; app, apparent; obs, obscured or a combination of these) and coupling constants (*J*, measured in Hertz (Hz) and quoted to the nearest 0.5 Hz) were identified using the commercially available iNMR 3.4.7 processor software. Where possible and appropriate, *J* values have been adjusted to match for coupling nuclei.

Assignment was based on chemical shift, integration, multiplicity, coupling constants and where appropriate, COSY, HMQC and HMBC experiments or by analogy to fully interpreted spectra for related compounds.

Carbon magnetic resonance spectra were recorded by broadband proton spin decoupling at ambient probe temperatures using an internal deuterium lock on the following instruments: Bruker Avance 400 CRYO QNP (100 MHz), Bruker Avance 400 QNP (100 MHz), Bruker Avance 500 CRYO (125 MHz). Chemical shifts (δ_C) are quoted in parts per million (ppm) to the nearest 0.1 ppm downfield of trimethylsilane (δ_C = 0) and are referenced to the residual non-deuterated solvent peak as follows: $CDCl_3$, 77.2 ppm; d_6-DMSO, 39.5 ppm. Chemical shifts were identified using the commercially available iNMR 3.4.7 processor software. Assignment was based on chemical shift, DEPT editing and where appropriate, HMQC and HMBC experiments or by analogy to fully interpreted spectra for related compounds.

High resolution mass spectrometry (HRMS) measurements were recorded on a Bruker Bioapex 4.7e FTICR or a Micromass LCT Premier spectrometer. Mass values are quoted within the error limits of ± 5 ppm mass units. ESI refers to the electrospray ionisation technique.

Characterisation data

Diethyl 2-(3-nitrobenzyl)malonate

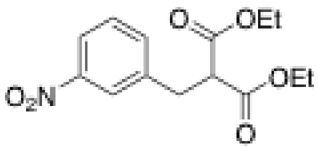

Adapted from the procedure of Rotthaus *et al.*[1] Diethyl malonate (4.42 mL, 29.1 mmol, 1 equiv) was dissolved in anhydrous THF (60 mL) and the resulting solution cooled to 0 °C. Sodium hydride (60% dispersion in mineral oil, 1.17 g, 29.1 mmol, 1 equiv) and 3-nitrobenzyl chloride (5.00 g, 29.1 mmol, 1 equiv) were added sequentially. The resulting mixture was refluxed o/n, allowed to cool to RT and poured into a sat. aqueous NH_4Cl solution. The organic layer was collected and the aqueous extracted with Et_2O (× 3). The organic fractions were combined, washed with water and brine, dried ($MgSO_4$) and concentrated *in vacuo*. The crude product was purified by column chromatography (SiO_2; pet ether-EtOAc gradient, 12:1-4:1) to yield the title compound as a light yellow oil (4.37 g, 14.8 mmol, 51%).

R_f (SiO_2; pet ether-EtOAc, 4:1) 0.40; **IR** ν_{max} (neat/cm^{-1}) 1724 s (C=O), 1528 s (NO_2), 1350 s (NO_2); **^{1}H NMR** (500 MHz; $CDCl_3$) δ_H 8.10-8.08 (2H, m, Phenyl CH and Phenyl CH), 7.57 (1H, d, *J* = 7.5 Hz, Phenyl CH), 7.48-7.45 (1H, m, Phenyl CH), 4.23-4.13 (4H, m, $OC\underline{H}_2CH_3$), 3.67 (1H, t, *J* = 8.0 Hz, $CH_2C\underline{H}$), 3.32 (2H, d, *J* = 8.0 Hz, $C\underline{H}_2CH$), 1.23 (6H, t, *J* = 7.0 Hz, $OCH_2C\underline{H}_3$); **^{13}C NMR** (125 MHz; $CDCl_3$) δ_C 168.4 (C=O), 148.5 (Phenyl C), 140.1 (Phenyl C), 135.4 (Phenyl CH), 129.6 (Phenyl CH), 124.0 (Phenyl CH), 122.1 (Phenyl CH), 62.0 ($O\underline{C}H_2CH_3$), 53.4 ($CH_2\underline{C}H$), 34.3 ($\underline{C}H_2CH$), 14.2 ($OCH_2\underline{C}H_3$); **HRMS** (ESI+) *m/z* found $[M+H]^+$ 296.1130, $C_{14}H_{18}NO_6^+$ required 296.1134.

Diethyl 2-(3-aminobenzyl)malonate

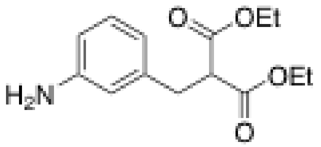

Diethyl 2-(3-nitrobenzyl)malonate (2.56 g, 8.68 mmol, 1 equiv) was dissolved in EtOAc (0.07 M) followed by the addition of platinum (IV) oxide (10 mol%) at RT. The resulting mixture was vigorously stirred under H_2 until TLC analysis indicated complete consumption of starting material (ninhydrin stain, approx. reaction time: 1 hr). The mixture was filtered over celite and concentrated *in vacuo*. The title compound was isolated as a colourless oil (2.32 g, 8.68 mmol, quant.) that was used without further purification.

$\mathbf{R}_{\boldsymbol{f}}$ (SiO_2; pet ether-EtOAc, 4:1) 0.08; **IR** ν_{max} (neat/cm^{-1}) 3466 w (NH_2), 3379 w (NH_2), 1723 s (C=O); **^{1}H NMR** (400 MHz; $CDCl_3$) δ_H 7.07-7.03 (1H, m, Phenyl CH), 6.60-6.58 (1H, m, Phenyl CH), 6.54-6.52 (2H, m, Phenyl CH and Phenyl CH), 4.22-4.10 (4H, m, $OC\underline{H}_2CH_3$), 3.61 (1H, obs t, *J* = 8.0 Hz, $CH_2C\underline{H}$), 3.61 (2H, obs br s, NH_2), 3.12 (2H, d, *J* = 8.0 Hz, $C\underline{H}_2CH$), 1.22 (6H, t, *J* = 7.0 Hz, $OCH_2C\underline{H}_3$); **^{13}C NMR** (100 MHz; $CDCl_3$) δ_C 169.1 (C=O), 146.6 (Phenyl C), 139.3 (Phenyl C), 129.5 (Phenyl CH), 119.1 (Phenyl CH), 115.7 (Phenyl CH), 113.6 (Phenyl CH), 61.6 ($O\underline{C}H_2CH_3$), 53.9 ($CH_2\underline{C}H$), 34.8 ($\underline{C}H_2CH$), 14.2 ($OCH_2\underline{C}H_3$); **HRMS** (ESI+) *m/z* found $[M+H]^+$ 266.1394, $C_{14}H_{20}NO_4^+$ required 266.1392.

Methyl thiophene-2-carboxylate

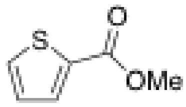

To a solution of 2-thiophenecarboxylic acid (10.0 g, 78.0 mmol, 1 equiv) in MeOH (100 mL) was added a concentrated solution of H_2SO_4 (5 mL). The resulting solution was heated to reflux for 17 hr, allowed to cool to RT and concentrated *in vacuo.* The residue was dissolved in EtOAc, washed with a sat. aqueous $NaHCO_3$ solution (× 3), dried ($MgSO_4$) and concentrated *in vacuo*. **111** was isolated as a brown oil (9.86 g, 69.4 mmol, 92%) that was used without further purification.

R*f* (SiO_2; pet ether-EtOAc, 4:1) 0.52; **IR** ν_{max} (neat/cm^{-1}) 1703 s (C=O); **^{1}H NMR** (400 MHz; $CDCl_3$) δ_H 7.80 (1H, dd, *J* = 4.0 and 1.5 Hz, Thienyl CH), 7.55 (1H, dd, *J* = 5.0 and 1.5 Hz, Thienyl CH), 7.10 (1H, dd, *J* = 5.0 and 4.0 Hz, Thienyl CH), 3.89 (3H, s, OCH_3); **^{13}C NMR** (100 MHz; $CDCl_3$) δ_C 162.8 (C=O), 133.7 (Thienyl C), 133.6 (Thienyl CH), 132.4 (Thienyl CH), 127.9 (Thienyl CH), 52.3 (OCH_3); **HRMS** (ESI+) *m/z* found $[M+H]^+$ 143.0172, $C_6H_7O_2S^+$ required 143.0167.

^{1}H and ^{13}C NMR data consistent with that previously reported.[2]

3-Iodo-*N*-methoxy-*N*-methylbenzamide

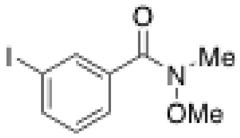

1,1′-carbonyldiimidazole (3.39 g, 20.9 mmol, 1.3 equiv) was added to a stirring solution of 3-iodobenzoic acid (4.00 g, 16.1 mmol, 1 equiv) in anhydrous THF (22 mL) and the resulting mixture stirred for 2 hr at RT. *N*,*O*-dimethylhydroxylamine hydrochloride (1.57 g, 16.1 mmol, 1 equiv) was added and the mixture stirred for 24 hr. The reaction was quenched with a sat. aqueous $NaHCO_3$ solution. The organic layer was collected and the aqueous extracted with Et_2O (× 3). The organic fractions were combined, washed with a 10% aqueous HCl solution, dried ($MgSO_4$) and concentrated *in vacuo*. The crude product was purified by column chromatography (SiO_2; pet ether-EtOAc, 4:1) to yield the title compound as a light yellow oil (3.12 g, 10.7 mmol, 67%).

R*f* (SiO_2; pet ether-EtOAc, 4:1) 0.19; **IR** ν_{max} (neat/cm^{-1}) 1636 s (C=O); **^{1}H NMR** (400 MHz; $CDCl_3$) δ_H 8.01-8.00 (1H, m, Phenyl CH), 7.79-7.76 (1H, m, Phenyl CH), 7.65-7.62 (1H, m, Phenyl CH), 7.14 (1H, app dt, *J* = 8.0 and 1.0 Hz, Phenyl CH), 3.54 (3H, app d, *J* = 1.0 Hz, OCH_3), 3.34 (3H, app d, *J* = 1.0 Hz, NCH_3); **^{13}C NMR** (100 MHz; $CDCl_3$) δ_C 168.5 (C=O^{Amide}), 139.9 (Phenyl CH), 137.4 (Phenyl CH), 136.4 (Phenyl C), 130.2 (Phenyl CH), 127.8 (Phenyl CH), 94.0 (Phenyl C), 61.4 (OCH_3), 34.0 (NCH_3); **HRMS** (ESI+) *m*/*z* found $[M+H]^+$ 291.9836, $C_9H_{11}NO_2I^+$ required 291.9834.

Methyl 5-(3-iodobenzoyl)thiophene-2-carboxylate

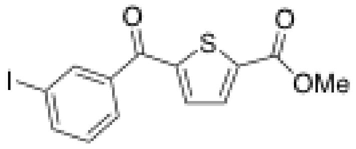

Methyl thiophene-2-carboxylate (831 mg, 5.84 mmol, 1 equiv) was dissolved in anhydrous THF (0.1 M) and the resulting solution cooled to -78 °C. Lithium diisopropylamide (2 M in THF/heptane/ethylbenzene; 1.2 equiv) was added dropwise and the resulting solution stirred at -78 °C for 15 min. 3-Iodo-*N*-methoxy-*N*-methylbenzamide (2.17 g, 5.84 mmol, 1 equiv) in anhydrous THF (0.1 M) at -78 °C was transferred into the reaction mixture *via* cannula. The resulting mixture was stirred at -78 °C for 1 hr, allowed to warm to RT and stirred for 2 hr. The reaction was quenched with a 10% aqueous HCl solution, the organic layer separated and the aqueous extracted with EtOAc (× 3). The organic fractions were combined, dried ($MgSO_4$) and concentrated *in vacuo*. The crude product was purified by column chromatography (SiO_2; pet ether-EtOAc, 10:1) to yield the title compound as a light yellow solid (409 mg, 1.10 mmol, 19%).

R*f* (SiO_2; pet ether-EtOAc, 4:1) 0.45; **mp** 119-122 °C (pet ether-EtOAc, 10:1); **IR** ν_{max} (neat/cm^{-1}) 1727 s (C=O^{Ester}), 1627 s (C=O^{Ketone}); **^{1}H NMR** (400 MHz; $CDCl_3$) δ_H 8.18 (1H, app t, *J* = 1.5 Hz, Phenyl CH), 7.95 (1H, ddd, *J* = 7.5, 1.5 and 1.0 Hz, Phenyl CH), 7.83-7.80 (2H, m, Thienyl CH and Phenyl CH), 7.58 (1H, d, *J* = 4.0 Hz, Thienyl CH), 7.26 (1H, obs app t, *J* = 7.5 Hz, Phenyl CH), 3.94 (3H, s, OCH_3); **^{13}C NMR** (100 MHz; $CDCl_3$) δ_C 186.5 (C=O^{Ketone}), 162.1 (C=O^{Ester}), 147.3 (Thienyl C), 141.8 (Phenyl CH), 140.5 (Thienyl C), 139.2 (Phenyl C), 138.1 (Phenyl CH), 134.2 (Thienyl CH), 133.3 (Thienyl CH), 130.4 (Phenyl CH), 128.5 (Phenyl CH), 94.4 (Phenyl C), 52.9 (OCH_3); **HRMS** (ESI+) *m/z* found $[M+H]^+$ 372.9404, $C_{13}H_{10}O_3SI^+$ required 372.9395.

5-(3-Iodobenzoyl)thiophene-2-carboxylic acid

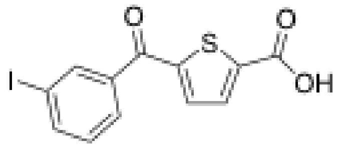

To a stirring solution of methyl 5-(3-iodobenzoyl)thiophene-2-carboxylate (319 mg, 0.86 mmol, 1 equiv) in a THF-H_2O (4:1) solution (0.0125 M) was added LiOH.H_2O (4 equiv) at RT. When TLC analysis indicated complete consumption of ester, the solution was concentrated *in vacuo*. The residue was suspended in the minimum amount of H_2O, acidified to pH 1 with a 10% aqueous HCl solution and extracted with EtOAc (× 3). The organic fractions were combined and extracted with a sat. aqueous $NaHCO_3$ solution (× 3). The aqueous solution was re-acidified to pH 1 with a 10% aqueous HCl solution and re-extracted with EtOAc (× 3). The organic fractions were combined, dried ($MgSO_4$), concentrated *in vacuo* and lyophilised in a MeCN-H_2O (1:1) solution to yield the title compound as a cream solid (285 mg, 0.80 mmol, 93%) that was used without further purification

mp 214-215 °C (EtOAc); **IR** ν_{max} (neat/cm^{-1}) 3359-2352 br (OH), 1674 s (C=O), 1627 s (C=O); **^{1}H NMR** (500 MHz; d_6-DMSO) δ_H 8.11 (1H, app t, J = 2.0 Hz, Phenyl CH), 8.06 (1H, ddd, J = 8.0, 2.0 and 1.0 Hz, Phenyl CH), 7.86 (1H, ddd, J = 8.0, 2.0 and 1.0 Hz, Phenyl CH), 7.77 (1H, d, J = 4.0 Hz, Thienyl CH), 7.70 (1H, d, J = 4.0 Hz, Thienyl CH), 7.38 (1H, app t, J = 8.0 Hz, Phenyl CH); **^{13}C NMR** (125 MHz; d_6-DMSO) δ_C 186.3 (C=O^{Ketone}), 162.4 (C=O^{Acid}), 146.1 (Thienyl C), 142.2 (Thienyl C), 141.4 (Phenyl CH), 138.7 (Phenyl C), 137.1 (Phenyl CH), 135.6 (Thienyl CH), 133.4 (Thienyl CH), 130.9 (Phenyl CH), 128.5 (Phenyl CH), 95.2 (Phenyl C); **HRMS** (ESI+) *m/z* found $[M+H]^+$ 358.9216, $C_{12}H_8O_3SI^+$ required 358.9233.

Diethyl-2-(3-(5-(3-iodobenzoyl)thiophene-2-carboxamido)benzyl)malonate

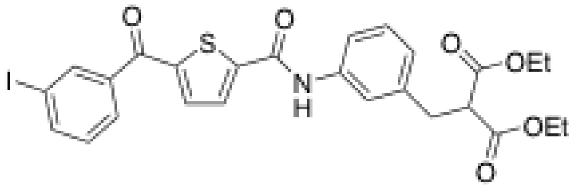

To a stirring ice-cold suspension of diethyl 2-(3-aminobenzyl)malonate (203 mg, 0.77 mmol, 1.24 equiv) and 5-(3-iodobenzoyl)thiophene-2-carboxylic acid (222 mg, 0.62 mmol, 1 equiv) in EtOAc (0.07 M) was added *N,N*-diisopropylethylamine (2 equiv) and propylphosphonic anhydride (50% solution in EtOAc, 1.6 equiv). The resulting solution was stirred at 0 °C for 30 min, allowed to warm to RT and stirred o/n. The reaction was quenched with H_2O and extracted with EtOAc (× 3). The organic fractions were combined, washed with a 10% aqueous HCl solution (× 3), a sat. aqueous $NaHCO_3$ solution (× 3), dried ($MgSO_4$) and concentrated *in vacuo* to furnish the title compound as a light yellow oil (173 mg, 0.29 mmol, 46%) that was used without further purification.

R*f* (SiO_2; pet ether-EtOAc, 2:1) 0.29; **IR** ν_{max} (neat/cm^{-1}) 3342 w (NH), 1728 s (C=O), 1642 s (C=O), 1611 m (C=O); **^{1}H NMR** (400 MHz; $CDCl_3$) δ_H 8.18 (1H, app t, *J* = 1.5 Hz, Phenyl CH), 7.96-7.94 (2H, m, NH and Phenyl CH), 7.83 (1H, ddd, *J* = 8.0, 1.5 and 1.0 Hz, Phenyl CH), 7.67 (1H, d, *J* = 4.0 Hz, Thienyl CH), 7.61 (1H, d, *J* = 4.0 Hz, Thienyl CH), 7.53-7.51 (1H, m, Phenyl CH), 7.48 (1H, app t, *J* = 2.0 Hz, Phenyl CH), 7.29-7.24 (2H, m, Phenyl CH and Phenyl CH), 7.03-7.01 (1H, m, Phenyl CH), 4.21-4.13 (4H, m, $OC\underline{H}_2CH_3$), 3.66 (1H, t, *J* = 7.5 Hz, $CH_2C\underline{H}$), 3.21 (2H, d, *J* = 7.5 Hz, $C\underline{H}_2CH$), 1.22 (6H, t, *J* = 7.0 Hz, $OCH_2C\underline{H}_3$); **^{13}C NMR** (100 MHz; $CDCl_3$) δ_C 186.8 (C=O^{Ketone}), 169.3 (C=O^{Ester}), 159.5 (C=O^{Amide}), 146.7 (Thienyl C), 146.3 (Thienyl C), 142.2 (Phenyl CH), 139.7 (Phenyl C), 139.6 (Phenyl C), 138.4 (Phenyl CH), 137.9 (Phenyl C), 135.0 (Thienyl CH), 130.8 (Phenyl CH), 129.9 (Phenyl CH), 129.4 (Thienyl CH), 128.9 (Phenyl CH), 126.2 (Phenyl CH), 121.3 (Phenyl CH), 119.4 (Phenyl CH), 94.8 (Phenyl C), 62.1 ($O\underline{C}H_2CH_3$), 54.2 ($CH_2\underline{C}H$), 35.1 ($\underline{C}H_2CH$), 14.6 ($OCH_2\underline{C}H_3$); **HRMS** (ESI+) *m/z* found $[M+H]^+$ 606.0449, $C_{26}H_{25}NO_6SI^+$ required 606.0447.

2-(3-(5-(3-Iodobenzoyl)thiophene-2-carboxamido)benzyl)malonic acid

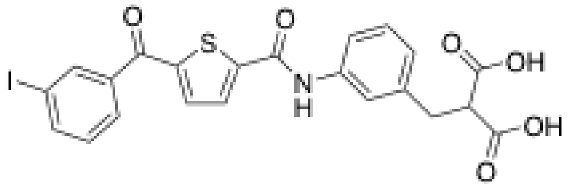

To a stirring solution of diethyl-2-(3-(5-(3-iodobenzoyl)thiophene-2-carboxamido)benzyl)malonate (97 mg, 0.16 mmol, 1 equiv) in a THF-H_2O (4:1) solution (0.0125 M) was added LiOH.H_2O (4 equiv) at RT. When TLC analysis indicated complete consumption of ester, the solution was concentrated *in vacuo*. The residue was suspended in the minimum amount of H_2O, acidified to pH 1 with a 10% aqueous HCl solution and extracted with EtOAc (× 3). The organic fractions were combined and extracted with a sat. aqueous $NaHCO_3$ solution (× 3). The aqueous solution was re-acidified to pH 1 with a 10% aqueous HCl solution and re-extracted with EtOAc (× 3). The organic fractions were combined, dried ($MgSO_4$), concentrated *in vacuo* and lyophilised in a MeCN-H_2O (1:1) solution to yield the title compound as a cream solid (46 mg, 0.084 mmol, 52%) that was used without further purification.

mp 101-103 °C (MeCN-H_2O, 1:1); **IR** ν_{max} (neat/cm^{-1}) 1713 m (C=O), 1638 m (C=O), 1611 s (C=O); **^{1}H NMR** (400 MHz; d_6-DMSO) δ_H 12.77 (2H, br s, COOH), 10.49 (1H, s, NH), 8.13 (1H, app t, *J* = 1.5 Hz, Phenyl CH), 8.12 (1H, d, *J* = 4.0 Hz, Thienyl CH), 8.07 (1H, ddd, *J* = 8.0, 1.5 and 1.0 Hz, Phenyl CH), 7.89 (1H, ddd, *J* = 8.0, 1.5 and 1.0 Hz, Phenyl CH), 7.80 (1H, d, *J* = 4.0 Hz, Thienyl CH), 7.64-7.61 (2H, m, Phenyl CH), 7.40 (1H, app t, *J* = 8.0 Hz, Phenyl CH), 7.29 (1H, app t, *J* = 7.5 Hz, Phenyl CH), 7.03 (1H, d, *J* = 7.5 Hz, Phenyl CH), 3.56 (1H, t, *J* = 7.5 Hz, $CH_2C\underline{H}$), 3.04 (2H, d, *J* = 7.5 Hz, $C\underline{H_2}CH$); **^{13}C NMR** (100 MHz; d_6-DMSO) δ_C 185.6 (C=O^{Ketone}), 169.5 (C=O^{Acid}), 158.3 (C=O^{Amide}), 146.8 (Thienyl C), 144.3 (Thienyl C), 140.7 (Phenyl CH), 138.6 (Phenyl C), 138.2 (Phenyl C), 137.6 (Phenyl C), 136.4 (Phenyl CH), 135.3 (Thienyl CH), 130.2 (Phenyl CH), 129.0 (Thienyl CH), 128.1 (Phenyl CH), 127.8 (Phenyl CH), 124.1 (Phenyl CH), 120.2 (Phenyl CH), 118.1 (Phenyl CH), 94.5 (Phenyl C), 52.8 ($CH_2\underline{C}H$), 33.7 ($\underline{C}H_2CH$); **HRMS** (ESI+) *m/z* found $[M+H]^+$ 549.9836, $C_{22}H_{17}NO_6SI^+$ required 549.9821.

References

1 O. Rotthaus, S. LeRoy, A. Tomas, K. M. Barkigia *et al.*, *Eur. J. Inorg. Chem.*, 2004, 1545-1551.

2 C. Liu, J. Wang, L. Meng, Y. Deng *et al.*, *Angew. Chem. Int. Ed.*, 2011, **50**, 5144-5148.

Supplementary Table S1. Oligonucleotides used in the study

Designation	Oligonucleotide sequence (5'-3')	Purpose
Plk1 Y421A Forward	G GTG GAC TAT TCG GAC AAG GCC GGC CTT GGG TAT CAG C	Site-directed mutagenesis of GFP-PLK1wt to generate Y421A
Plk1 Y421A Reverse	G CTG ATA CCC AAG GCC GGC CTT GTC CGA ATA GTC CAC C	Site-directed mutagenesis of GFP-PLK1wt to generate Y421A
Plk1 L478A Forward	CC TTG ATG AAG AAG ATC ACC GCC CTT AAA TAT TTC CGC	Site-directed mutagenesis of GFP-PLK1Y421A to incorporate L478A
Plk1 L478A Reverse	GCG GAA ATA TTT AAG GGC GGT GAT CTT CTT CAT CAA GG	Site-directed mutagenesis of GFP-PLK1Y421A to incorporate L478A
Plk1 L478A/ Y481D Forward	G AAG ATC ACC GCC CTT AAA GAT TTC CGC AAT TAC ATG AGC G	Site-directed mutagenesis of GFP-PLK1Y421A/L478A to incorporate Y481D

Plk1 L478A/ Y481D Reverse	C GCT CAT GTA ATT GCG GAA ATC TTT AAG GGC GGT GAT CTT C	Site-directed mutagenesis of GFP-PLK1Y421A/L478A to incorporate Y481D
Plk1 Forward	GCG CTC GAG ATG AGT GCT GCA G	Cloning Plk1 containing XhoI restriction site (underlined)
Plk1 Reverse	CTC GCG GCC GCT TAT TAG GAG GC	Cloning Plk1 containing NotI restriction site (underlined)
PBIP1 Forward	ATT GGA TCC ATG GCC CCG CGG GGG CGG CGG CGG	Cloning PBIP1 containing BamHI restriction site (underlined)
PBIP1 Reverse	GCC TCT AGA TCC CTG GTC AAG GAG CTT CTC TAA CTG	Cloning PBIP1 containing XbaI restriction site (underlined)
PBIP1 F71A Forward	GAA GAA ACT TAT GAG ACC GCT GAT CCT CCT TTA CAT AGC	Site-directed mutagenesis of PBIP1 to generate F71A
PBIP1 F71A Reverse	GCT ATG TAA AGG AGG ATC AGC GGT CTC ATA AGT TTC TTC	Site-directed mutagenesis of PBIP1 to generate F71A

PBIP1 T78A Forward	**ACC TTT GAT CCT CCT TTA CAT AGC GCA GCT ATA TAT GCT G**	Site-directed mutagenesis of PBIP1 to generate T78A
PBIP1 T78A Reverse	**CAG CAT ATA TAG CTG CGC TAT GTA AAG GAG GAT CAA AGG T**	Site-directed mutagenesis of PBIP1 to generate T78A

*Residues mutated for site-directed mutagenesis are highlighted in grey.

Supplementary Table S2. SiRNA sequences used in the study

Designation	Target sequence (5'-3')	Target	Manufacturer
siLuc	CGUACGCGGAAUACUUCGA	Luciferase (non-targeting control)	MWG
siPlk1	CAACGGCAGCGTGCAGATCAA	Plk1	Qiagen
siPlk1 3'UTR	CCATATGAATTGTACAGAATA	3'UTR of Plk1	Qiagen

Supplementary Table S3. Crystallographic data collection and refinement statistics

Protein	Plk1 PBD	Plk1 PBD
Ligand	Polotyrin	3-iodo benzyl bromide
PDB code	5NEI	5NMM
**Data collection**		
Synchrotron and beamline	DLS, i24	DLS, i03
Wavelength (Å)	0.9830	0.9200
Temperature (K)	100.0	100.0
**Data processing**		
Resolution (Å)	45.68-2.68 (2.75-2.68)	46.52-2.02 (2.07-2.02)
Space group	$P2_1$	$P2_1$
Unit cell: a,b,c (Å)	33.350, 91.360, 35.940	33.360, 93.040, 35.910
a,b,g (deg)	90.00, 99.71, 90.00	90.00, 100.22, 90.00
R_{merge}	0.072 (0.470)	0.058 (0.701)
R_{meas}	0.115 (0.704)	0.087 (0.877)
Total number of observations	15,152 (1214)	52232 (3925)
Total number unique	5965	13,900 (1009)
Mean((I)/s(I))	9.56 (2.19)	11.4 (2.4)
Completeness (%)	99.2 (99.6)	98.1 (97.3)
Multiplicity	2.6 (2.6)	3.8 (3.9)
**Refinement**		
Resolution (Å)	45.68-2.68(3.38-2.68)	46.52-2.02 (2.17-2.02)
R_{work}	0.199 (0.229)	0.192 (0.259)
R_{free}	0.248 (0.303)	0.237 (0.328)
No. of non-H atoms	1797	1763
Protein atoms	1755	1720
Ligand atoms	31	9
Waters	11	34
RMSD bonds (Å)	0.008	0.005
RMSD angles (deg)	1.126	0.868
Ramachandran favored (%)	94	95
Ramachandran allowed (%)	5	5
Ramachandran outliers (%)	1	0
Molprobity clashscore	17.1	10.9
Average B-factor (Å^2)	55.7	46.7
of macromolecules	55.7	46.7
of ligands	50.8	46.7

SUPPLEMENTARY FIGURE LEGENDS

S1. Inducible expression of GFP-PLK1$_{Wt/AAD/AM}$ after induction with doxycycline. HeLa cells expressing GFP-PLK1$_{Wt/AAD/AM}$ were either treated with Dox for 16, 24 and 48h (+), or untreated (-). Cell extracts were analysed by immunoblotting using GFP antibody for GFP-PLK1$_{Wt/AAD/AM}$ in panels A/B/C respectively; β-actin blot shows uniform loading across lanes.

S2: Knockdown of PLK1 with siRNA and concomitant expression of GFP-PLK1$_{Wt/AAD/AM}$. HeLa cells inducibly expressing GFP-PLK1$_{Wt/AAD/AM}$ were treated with Dox and concomitantly transfected with SiRNA's as specified (siLuc –non-targeting SiRNA or SiPLK1 3'UTR or SiPlk1, see table S2). Cells extracts after 24h and 48h of treatment were analysed by immunoblotting using PLK1 antibody. Ponceau S-treated membranes show comparable loading of the lysates on each membrane. Asterisks (*) show cross-reacting bands.

S3. Localisation of GFP-PLK1$_{Wt/AAD/AM}$ on kinetochores in prometaphase cells. (A) Representative maximal-intensity projection images of cells showing kinetochores (KT) in red, centrosomes (CENT) in white, GFP-PLK1$_{Wt/AAD/AM}$ in green and DNA in blue used for quantification of GFP-PLK1 intensity in Fig. S3 (B). The cell lines were treated with Dox (0.5 mg.ml^{-1}) for 7 h, fixed and stained with CREST antiserum, anti-Pericentrin and Hoechst 33342 and analysed by immunofluorescence microscopy for GFP signal in prometaphase cells. (**B**) Quantification of intensity ratios of GFP-PLK1$_{Wt/AAD/AM}$ on CREST-stained kinetochores (KT) normalized to the corresponding GFP-PLK1 expression in cells. Image analysis was done using CellProfiler. Data from each cell is represented as a hollow circle, horizontal line (red) indicates mean intensity ratio and error bars indicate ± S.D. Statistical analysis was done using non-parametric, Mann-Whitney two-tailed test with 95% confidence interval. ***$p<0.0029$.

S4. Mitotic index (MI) of GFP-PLK1$_{Wt/AAD/AM}$ cells after 24h treatment with siPlk1 3'UTR. Cells treated with siPlk1 3'UTR were fixed and stained as described in the methods section. MI is expressed as a percentage of phospho-histone H3 positive cells per 100 DAPI-stained nuclei counted. Each bar is a mean of three replicates (each replicate =2000 cells) ± S.E.M.

S5. Representative images of single mitotic GFP-PLK1$_{Wt}$ cell in the FRAP experiment. At the onset of the experiment (t=0 s), both centrosomes show localization of GFP signal; upon photobleaching (t=1.4 s) the signal disappears in one of the centrosomes (see arrow head) and gradually reappears (t=1.9 to 9.7 s). Scale bar, 3μm.

S6. Immunoprecipitation of GFP-PLK1Wt/AAD/AM with NEDD1. (A, B). HeLa cells expressing GFP-PLK1$_{Wt/AAD/AM}$ were synchronized in mitosis by double thymidine block and released as shown in the experimental schedule in **Fig. 4B**. The cell lysates were immunoprecipitated using GFP-Trap® beads to pull down GFP-PLK1$_{Wt/AAD/AM}$ and analysed by immunoblotting. (**C**). HeLa cells expressing GFP-PLK1$_{Wt/AAD/AM}$ were synchronized in mitosis with nocodazole. The cell lysates were immunoprecipitated using GFP-Trap® beads to pull down GFP-PLK1$_{Wt/AAD/AM}$ and analysed by immunoblotting.

S7. Reciprocal co-Immunoprecipitation (co-IP) of GFP-PLK1Wt/AAD/AM with PBIP1. Reciprocal co-IP of Figure 4C. PBIP1$_{Wt}$-V5 was transfected in to uninduced HeLa cells and cells expressing GFP-PLK1$_{Wt/AAD/AM}$. 24h later cells were harvested, GFP-Trap® was used to pull down GFP-PLK1Wt/AAD/AM from the lysates and co-immunoprecipitates were analysed by immunoblotting.

S8. Treatment with Polotyrin causes chromosome congression defects in mitotic cells. Representative images used for MI determination (see Fig. 5D) were collected on Cellomics ArrayScan with a 20x Planfluor objective × 0.4 NA; cells were stained with Hoechst 33342 and phospho-Histone H3 (shown in blue and green in the merged image respectively). Insets were digitally magnified to show chromosome congression in Polotyrin versus DMSO-treated cells.

Supplementary Video M1. GFP-PLK1Wt cells treated with Plk1 3'UTR siRNA were imaged at 5 min intervals and displayed at 10 frames per second.

Supplementary Video M2. GFP-PLK1AAD cells treated with Plk1 3'UTR siRNA were imaged at 5 min intervals and displayed at 10 frames per second.

Supplementary Video M3. GFP-PLK1AM cells treated with Plk1 3'UTR siRNA were imaged at 5 min intervals and displayed at 10 frames per second.

Figure S1: Inducible expression of GFP-PLK1$_{Wt/AAD/AM}$ after induction with doxycycline.

A.

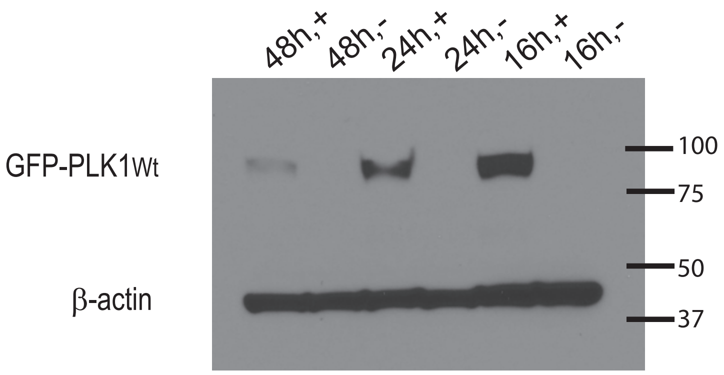

B.

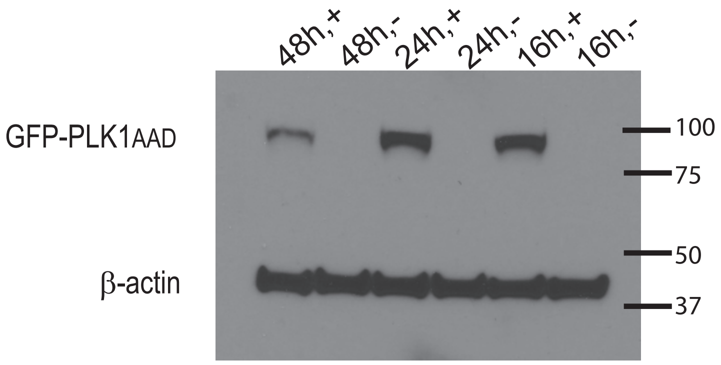

C.

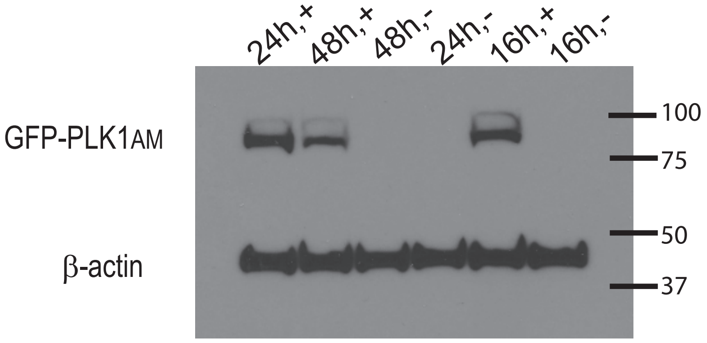

Figure S2: Knockdown of PLK1 with siRNA and concomitant expression of GFP-PLK1Wt/AAD/AM

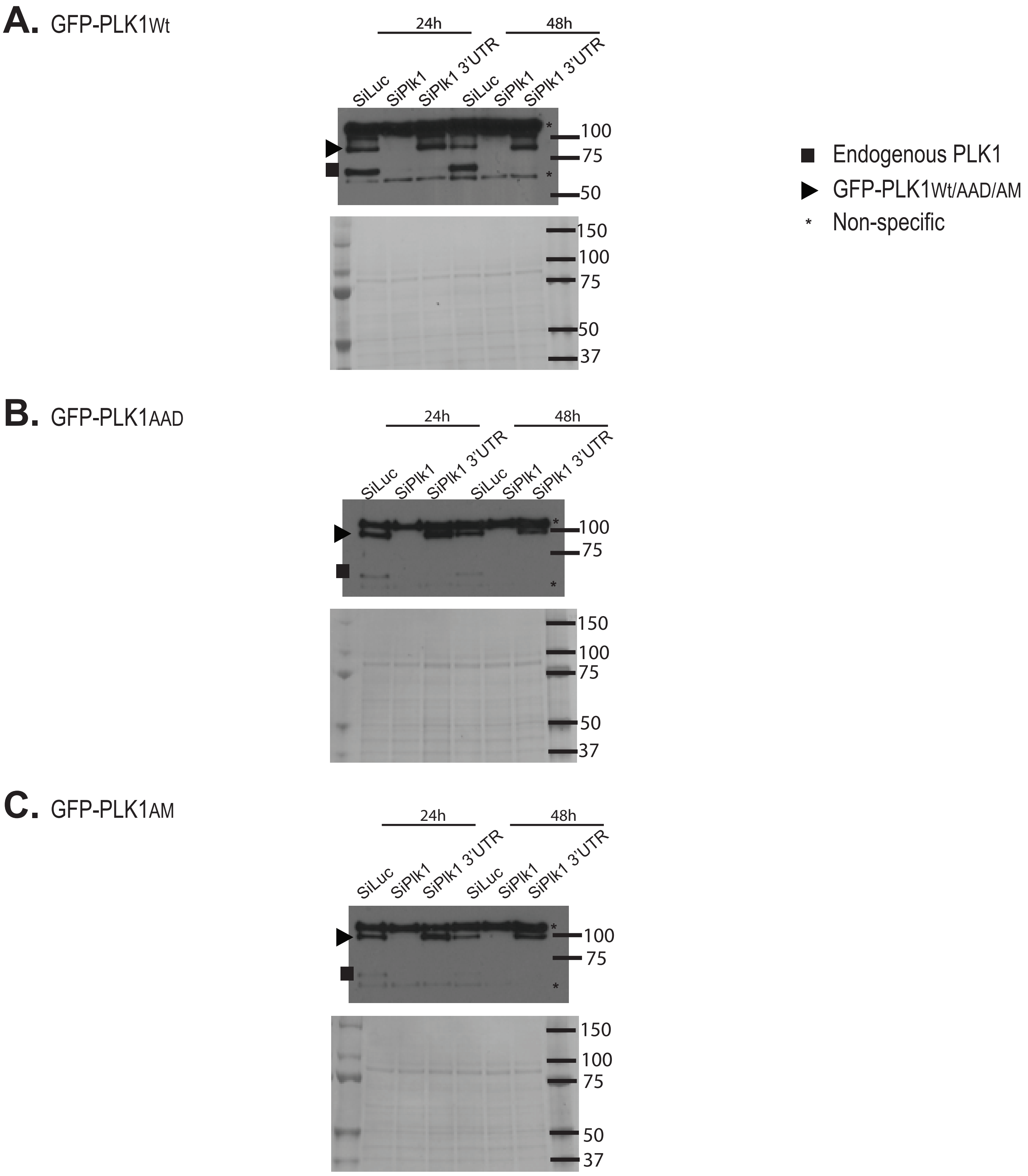

Figure S3: Localisation of GFP-PLK1Wt/AAD/AM on kinetochores in prometaphase cells

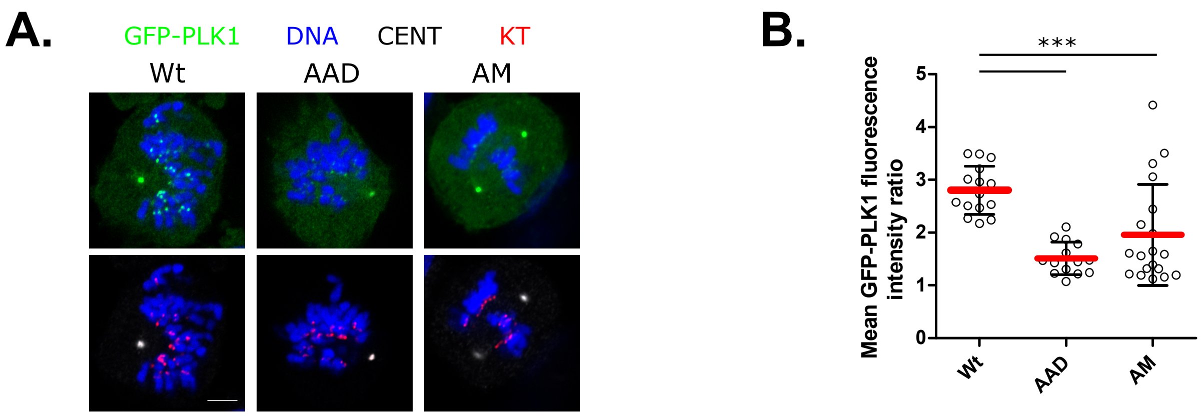

Figure S4: Mitotic index (MI) of GFP-PLK1Wt/AAD/AM after 24h of treatment with siPlk1 3'UTR

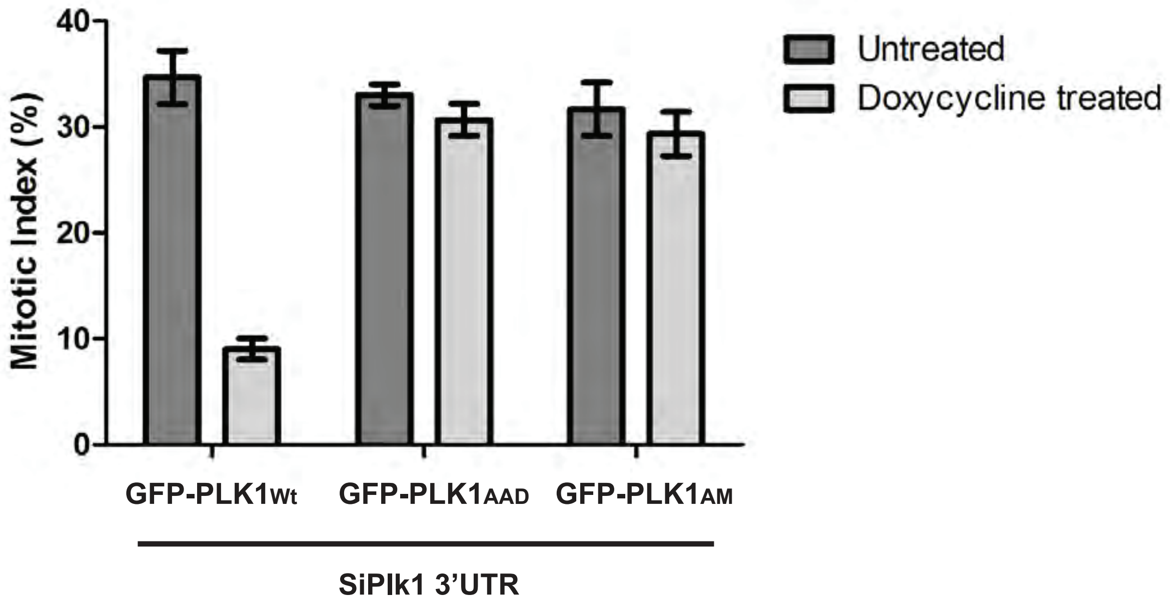

Figure S5: Representative images of single mitotic GFP-PLK1$_{Wt}$ cell in the FRAP experiment

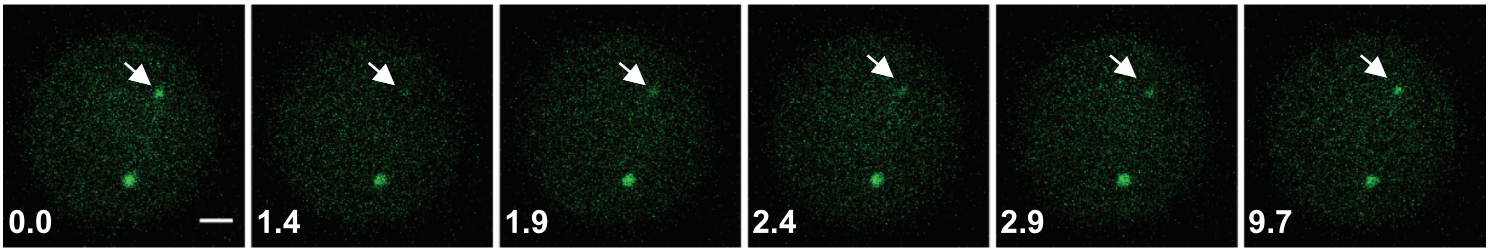

Figure S6: Immunoprecipitation of GFP-PLK1Wt/AAD/AM with NEDD1

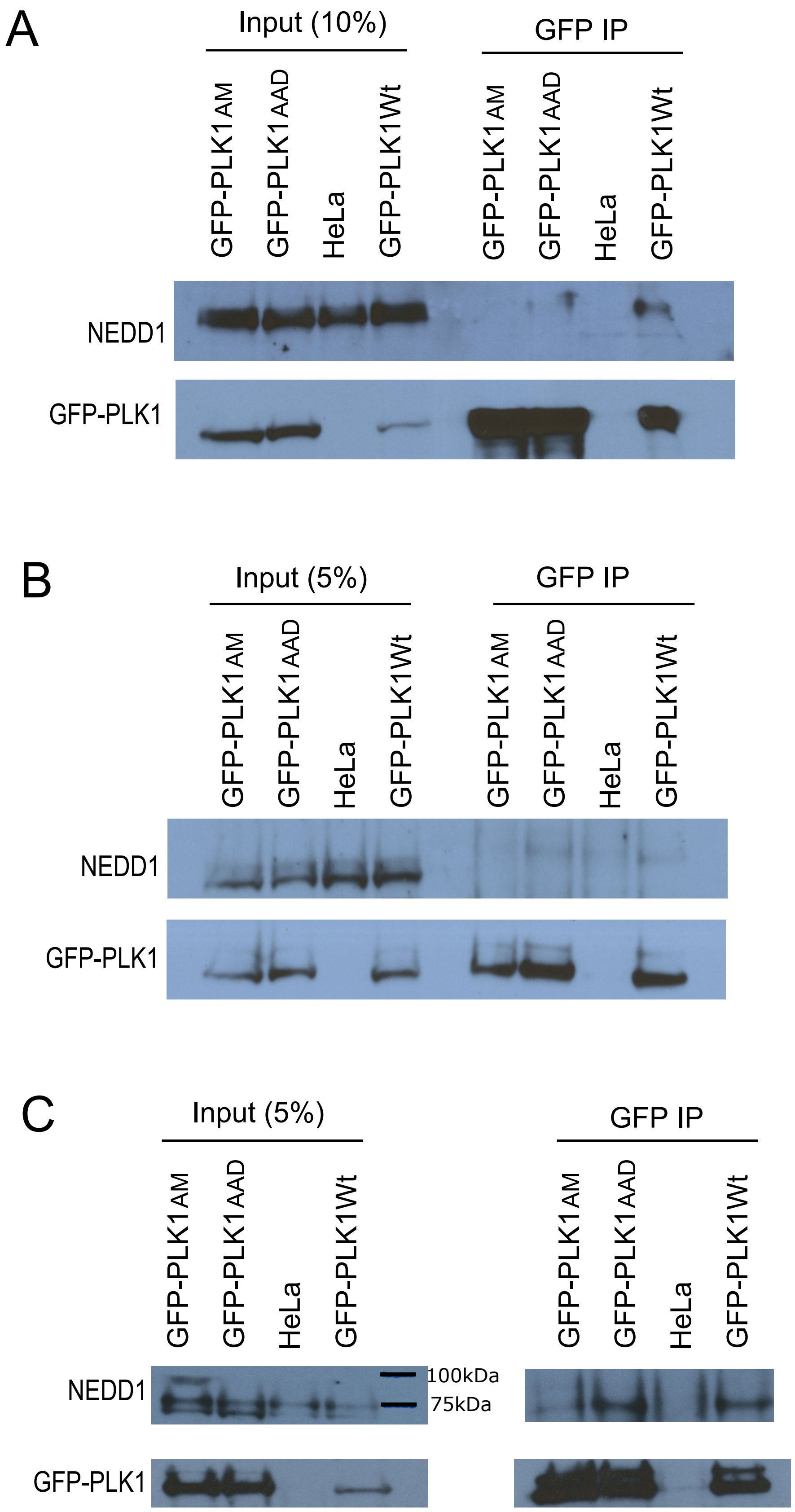

Figure S7: Reciprocal co-Immunoprecipitation (co-IP) of GFP-PLK1$_{Wt/AAD/AM}$ with PBIP1

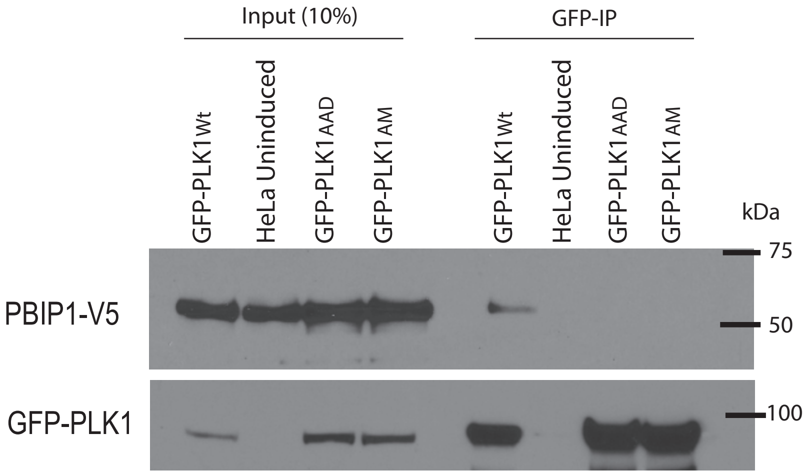

Figure S8: Treatment with Polotyrin causes chromosome congression defects in mitotic cells

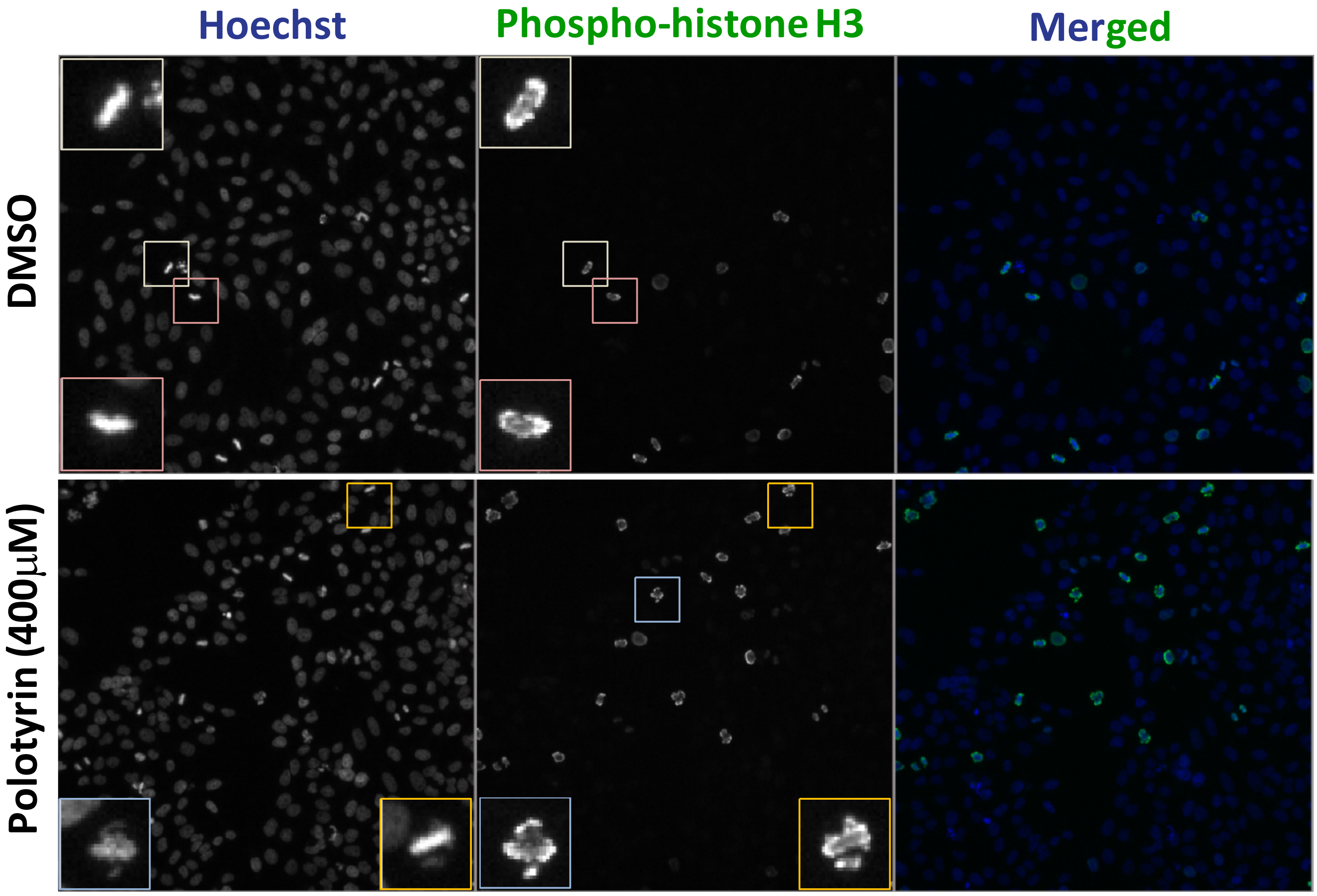

FULL SIZE BLOTS.
Boxed regions indicate area used in figures.
Figure 4B GFP

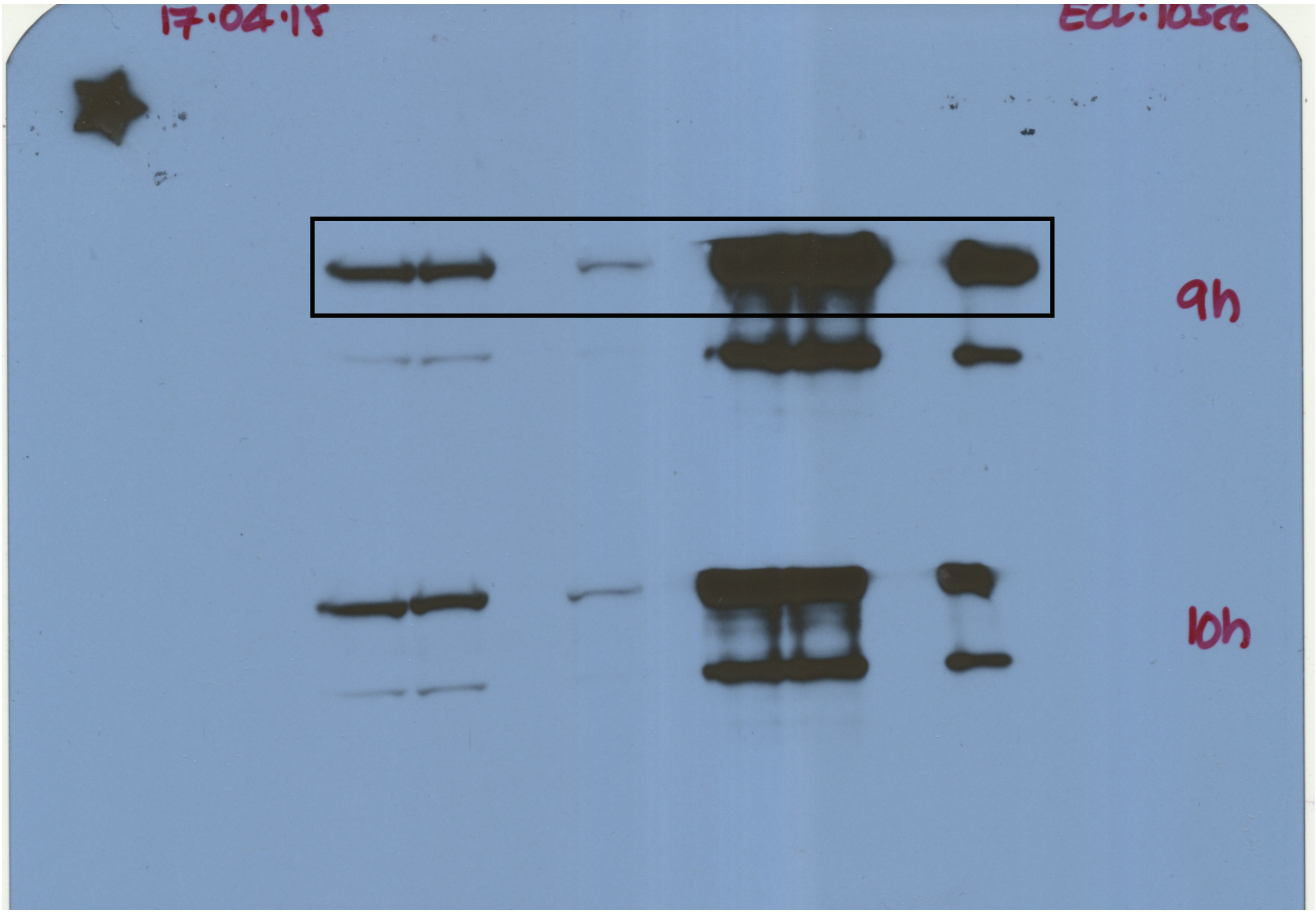

FULL SIZE BLOTS.
Boxed regions indicate area used in figures.
Figure 4B NEDD1 Short Exposure

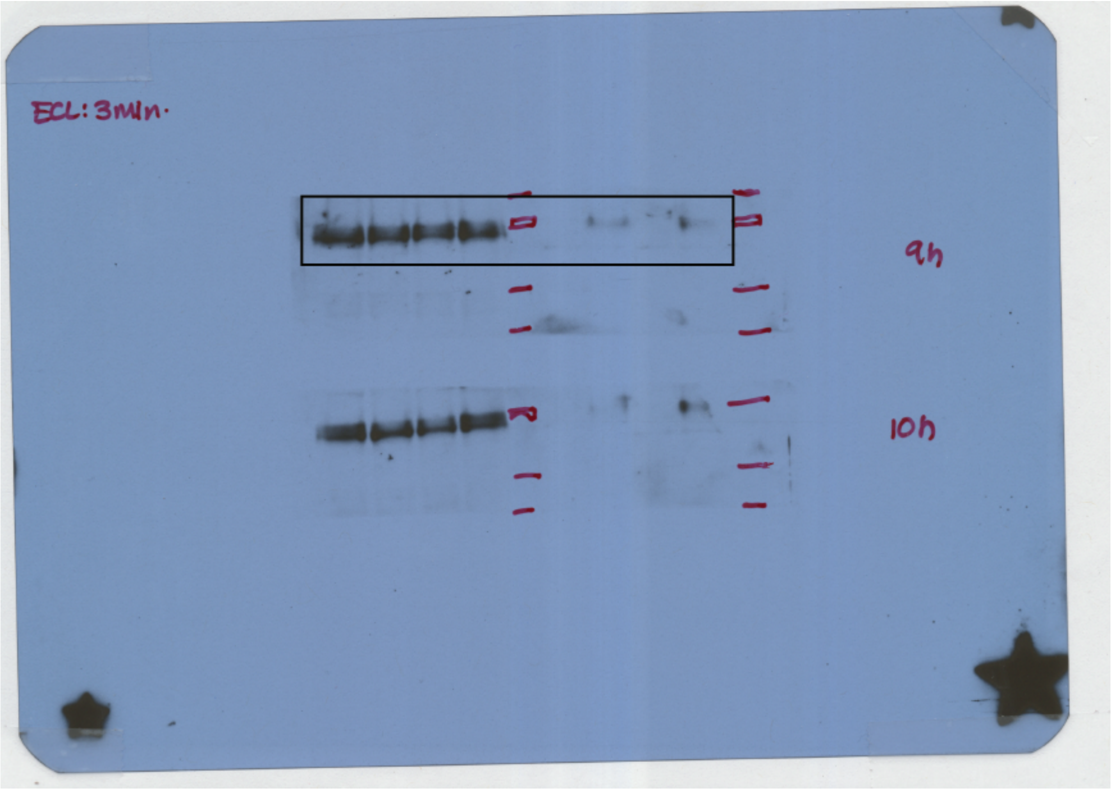

Figure 4B NEDD1 Long Exposure

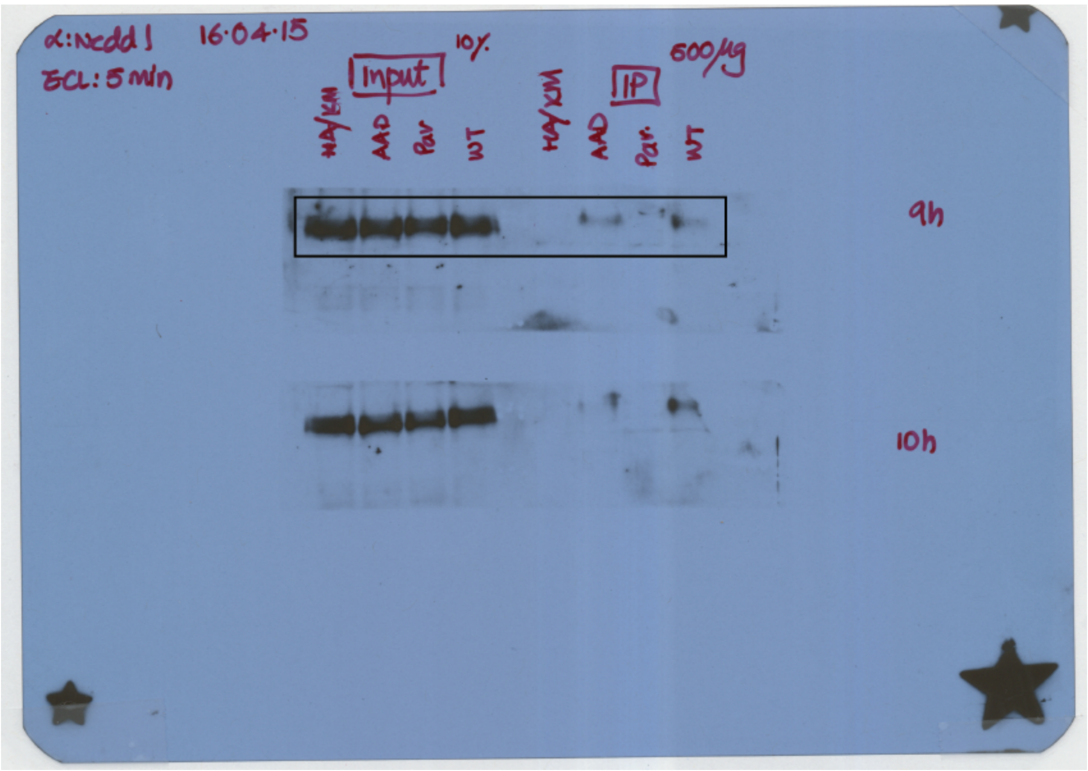

Figure 4C Long Exposure

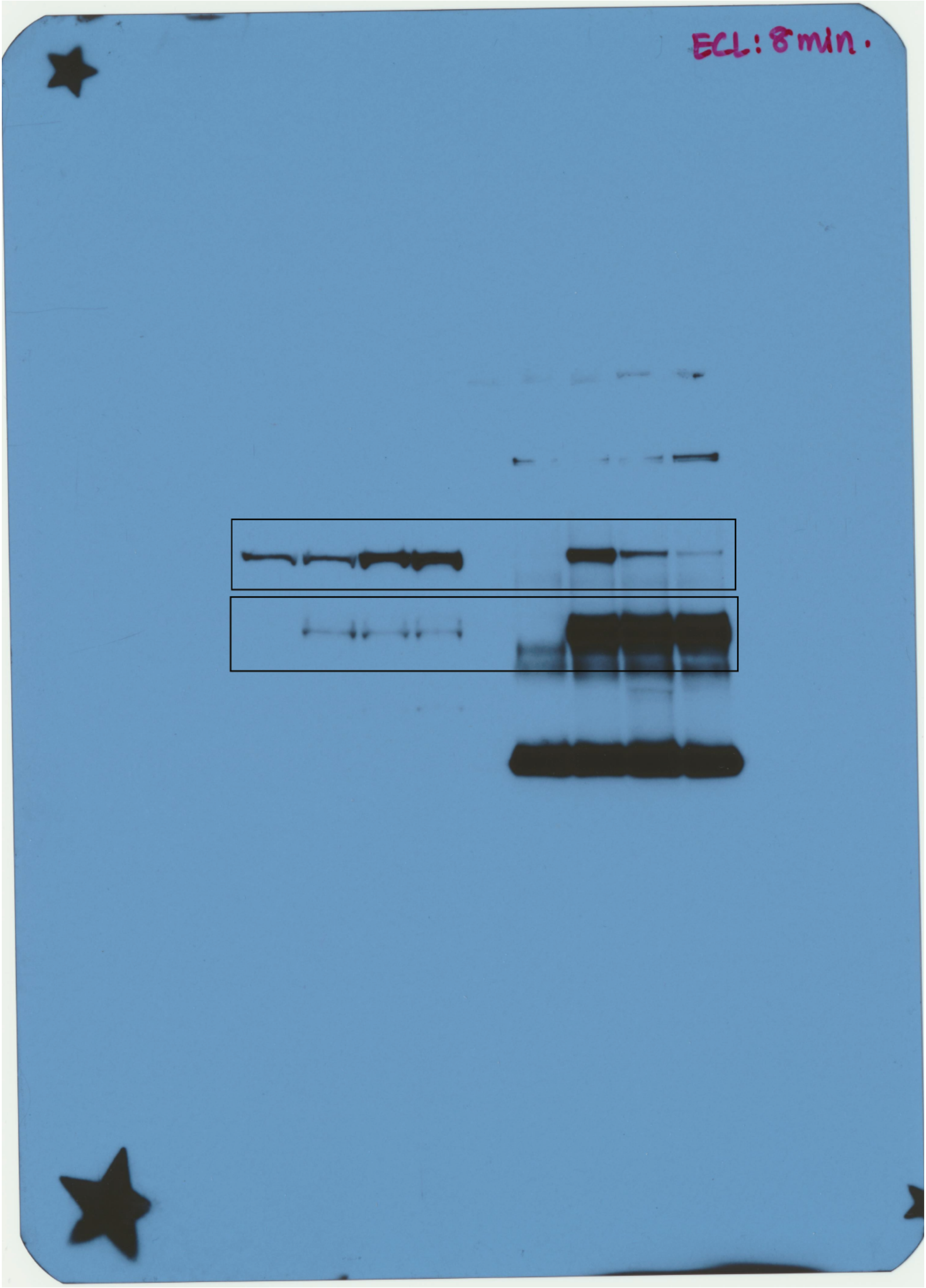

Figure 4C Short Exposure

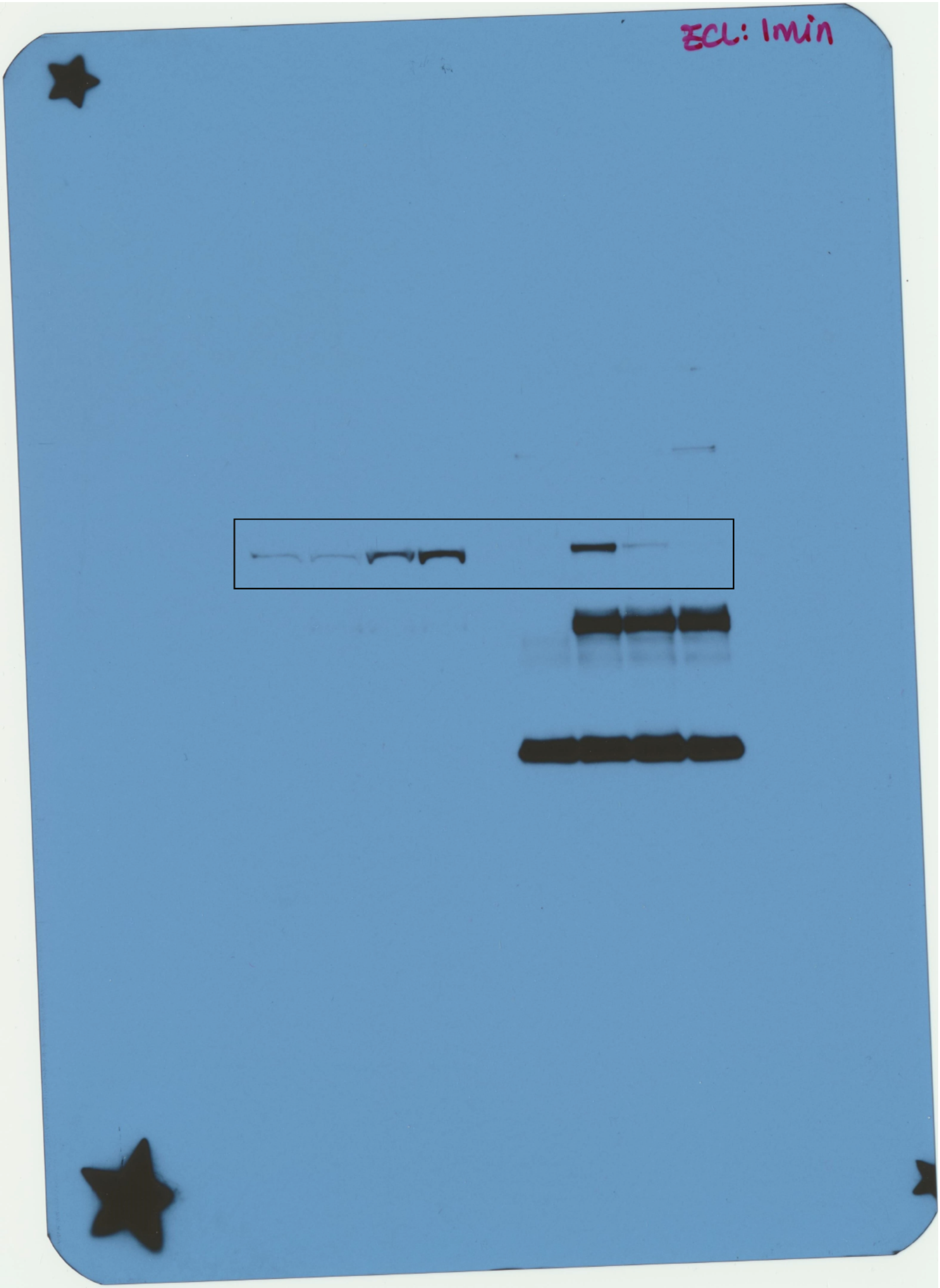

Figure 4D Long Exposure

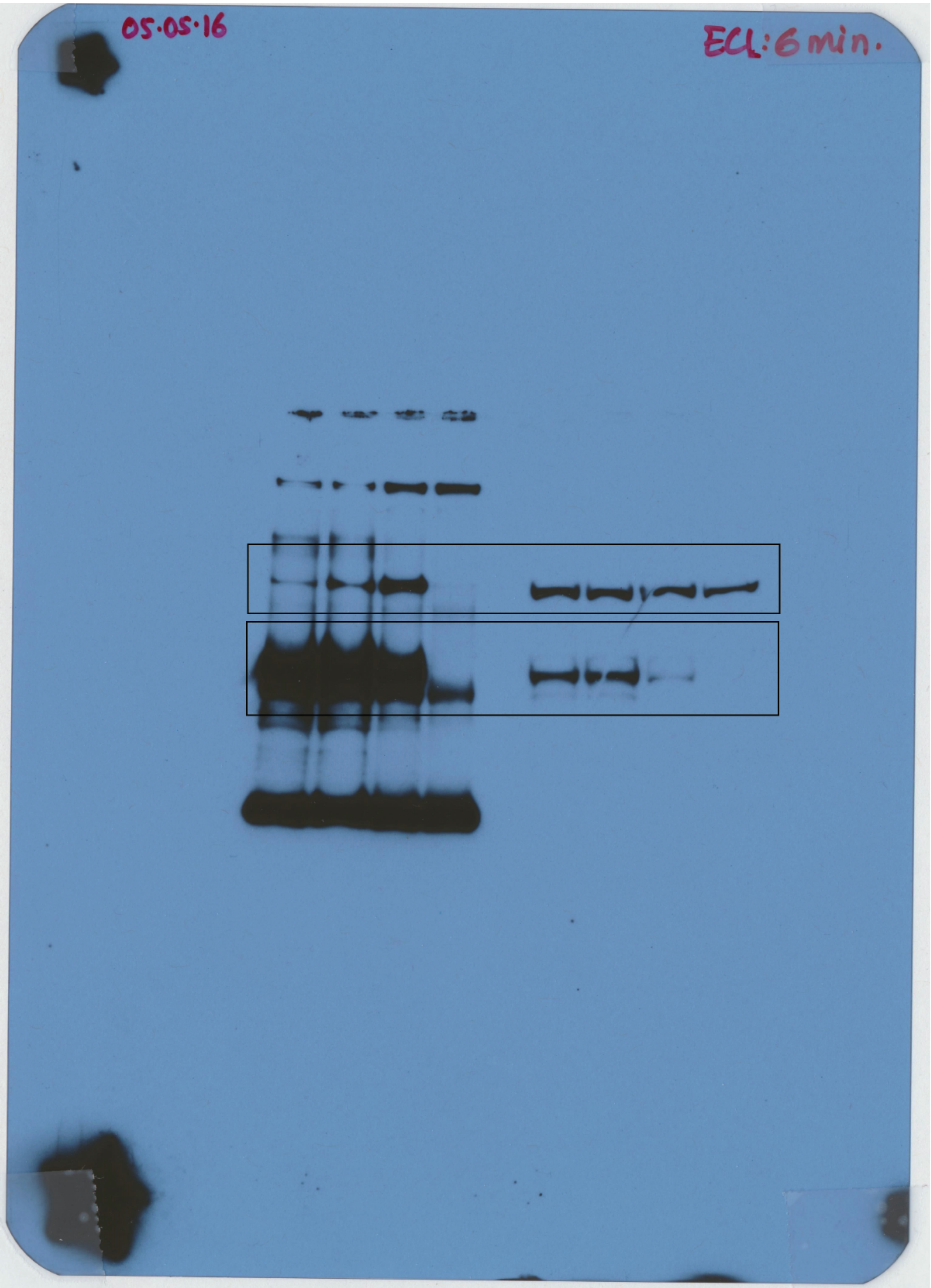

Figure 4D Short Exposure

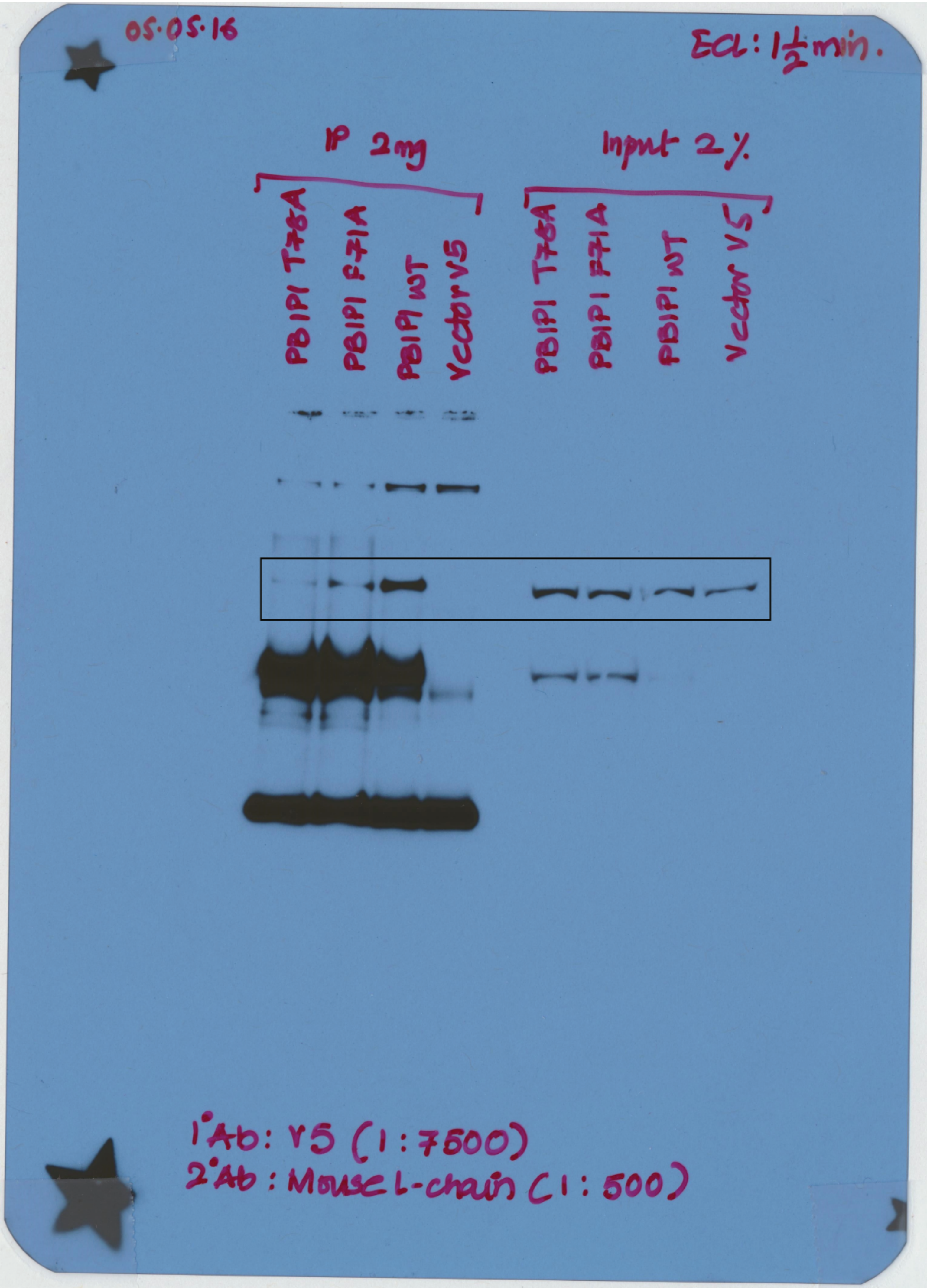

Figure 5F Long Exposure

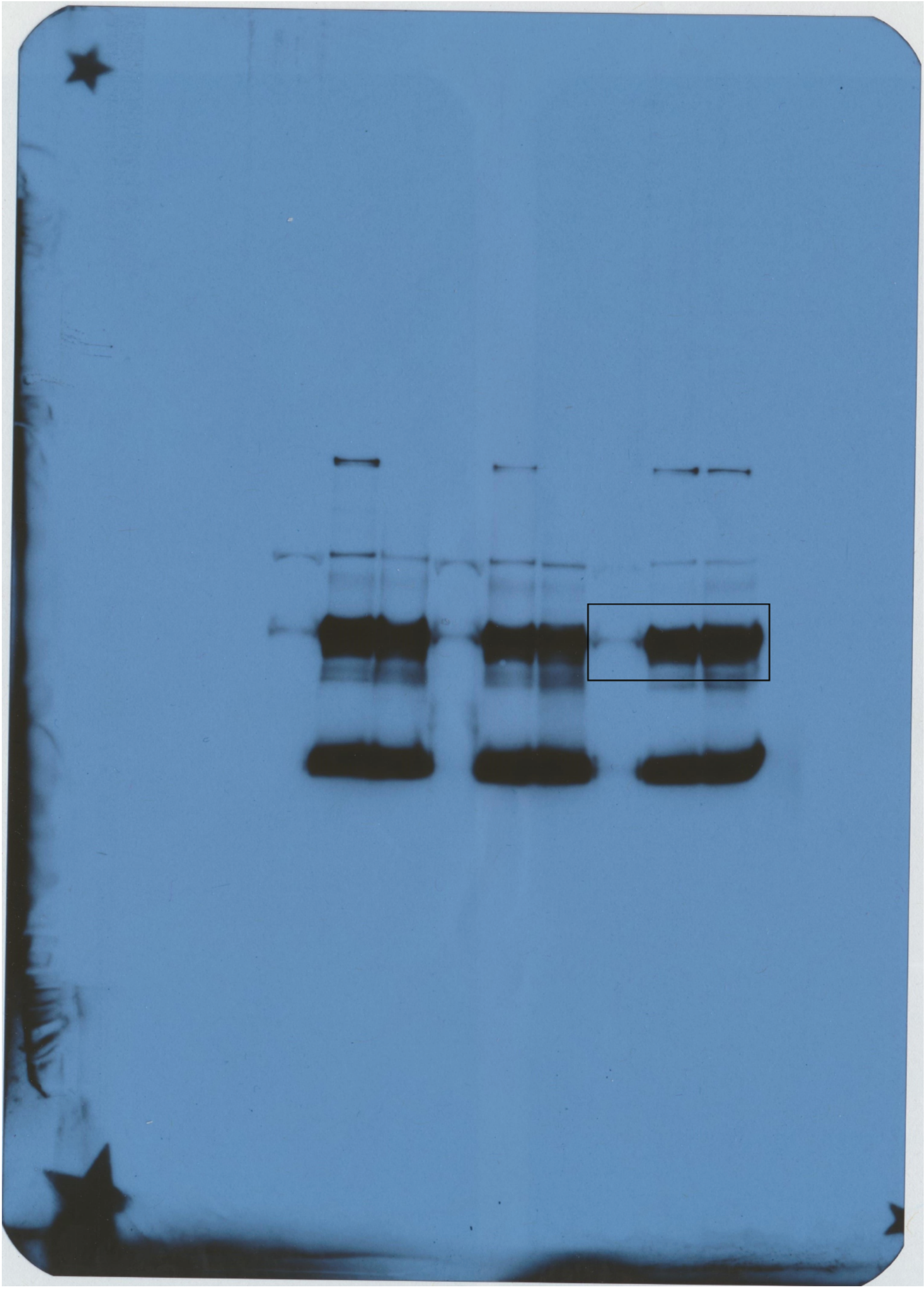

Figure 5F Short Exposure

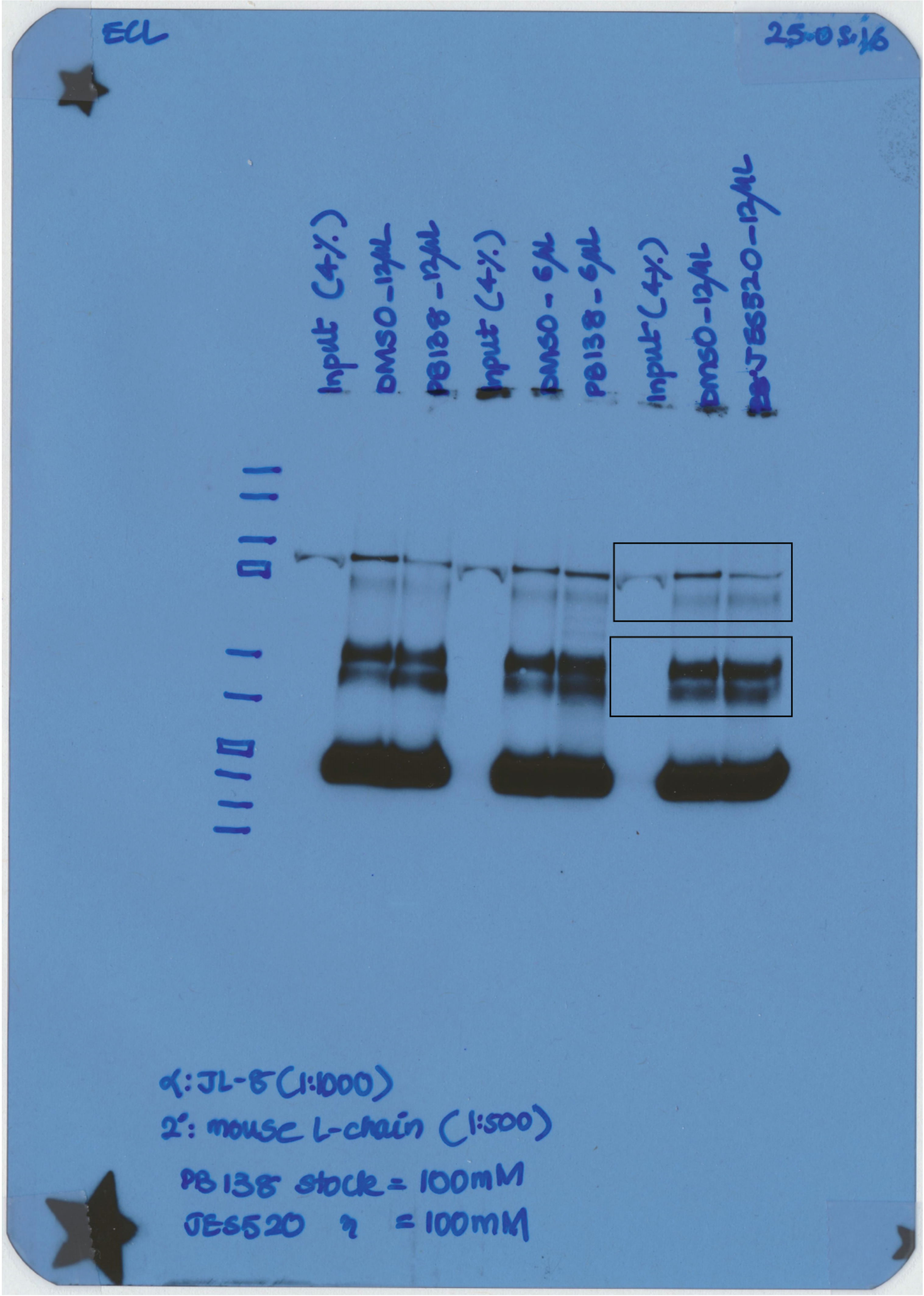

Figure S2 A

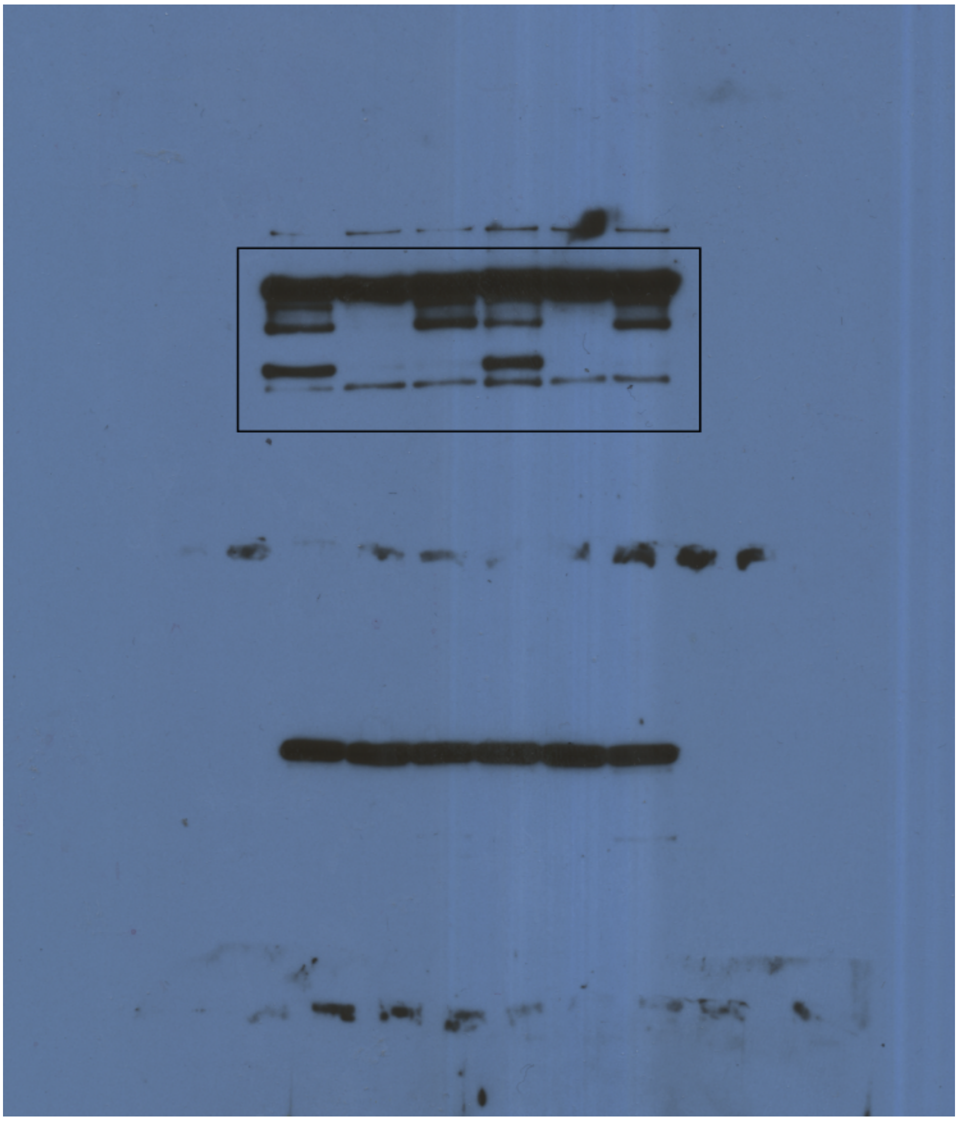

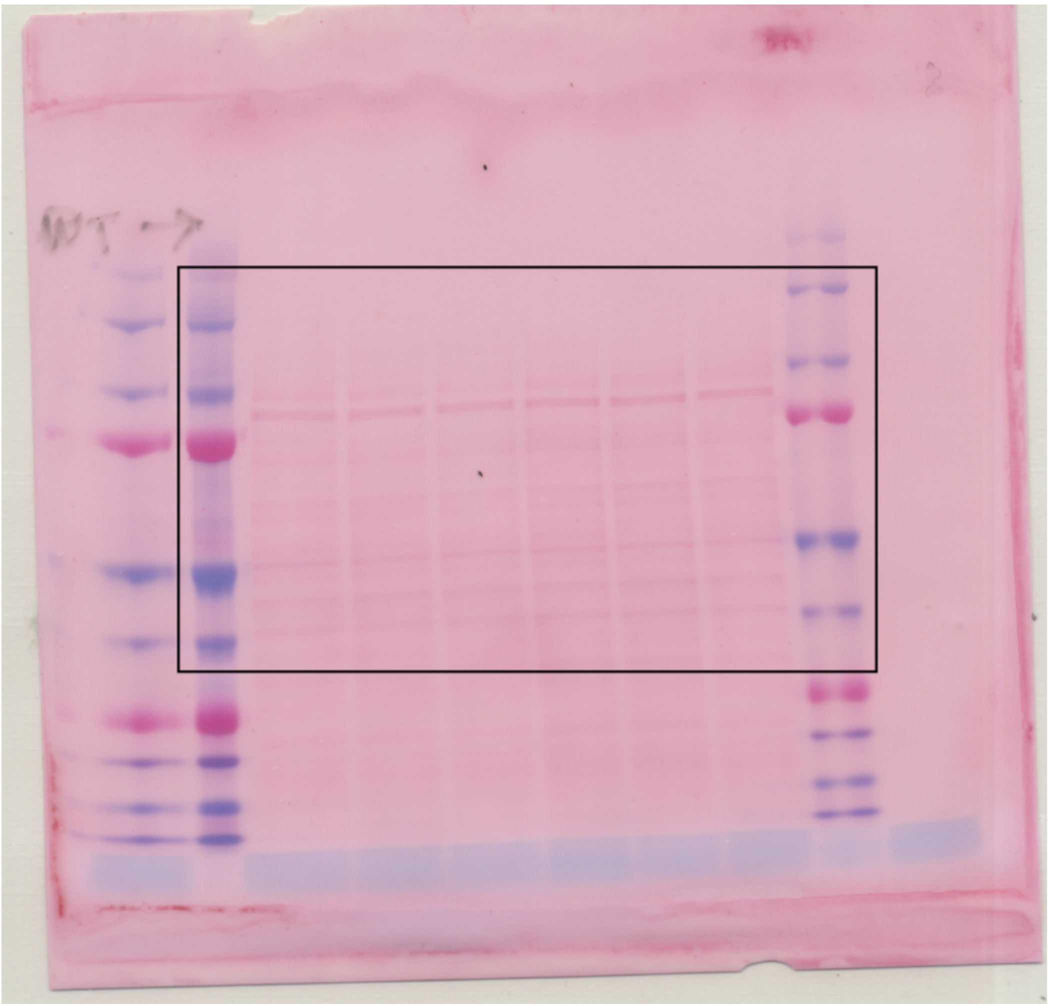

Figure S2 B

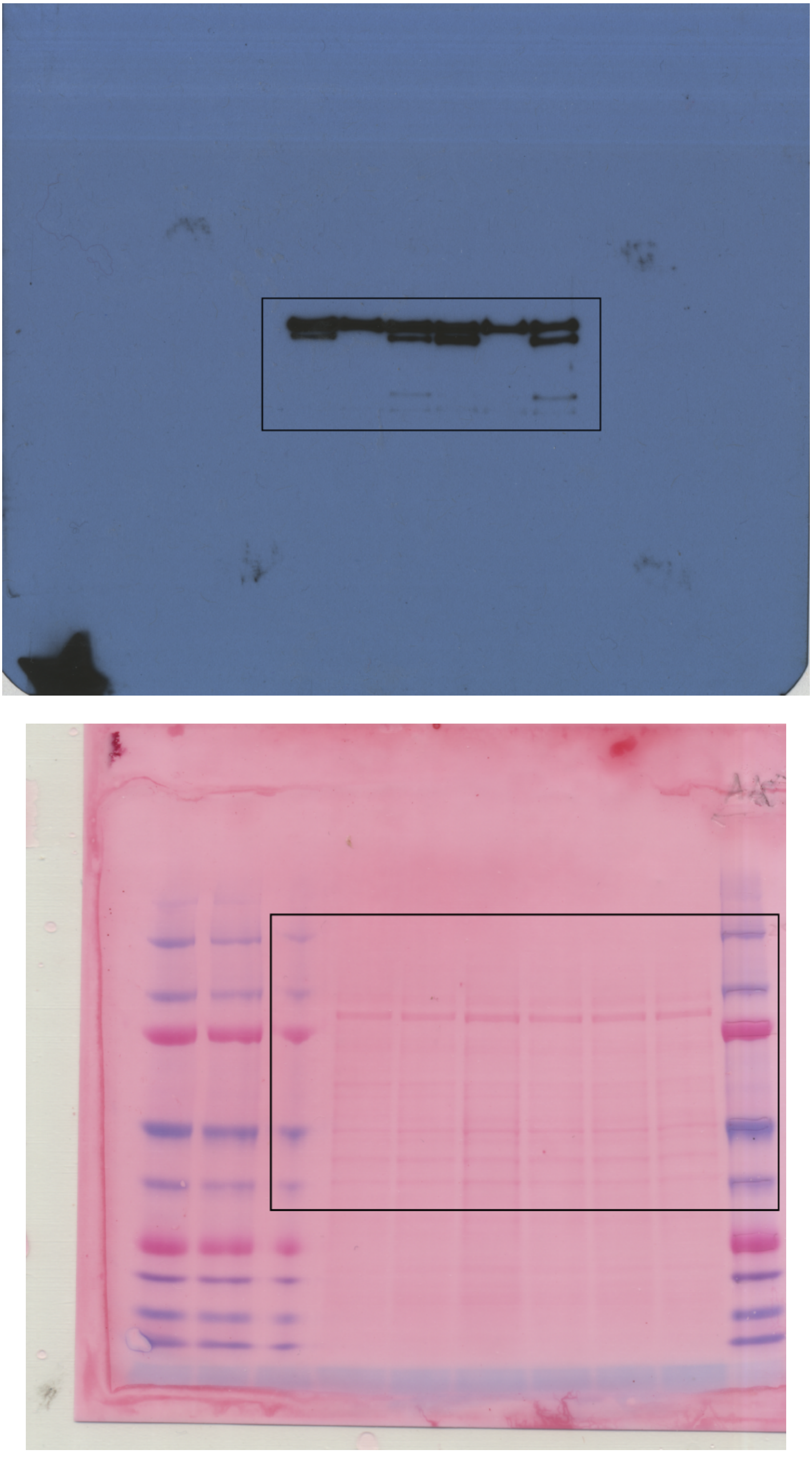

Figure S2 C

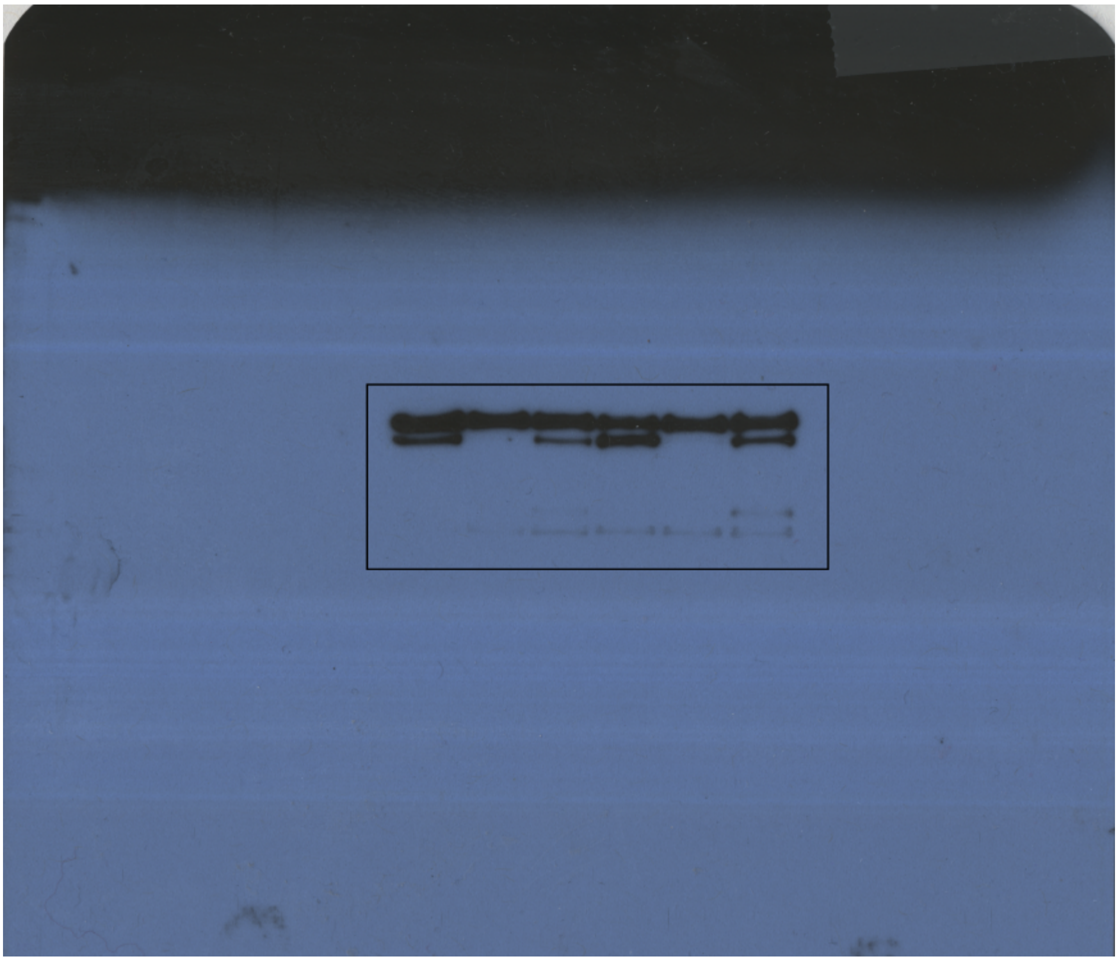

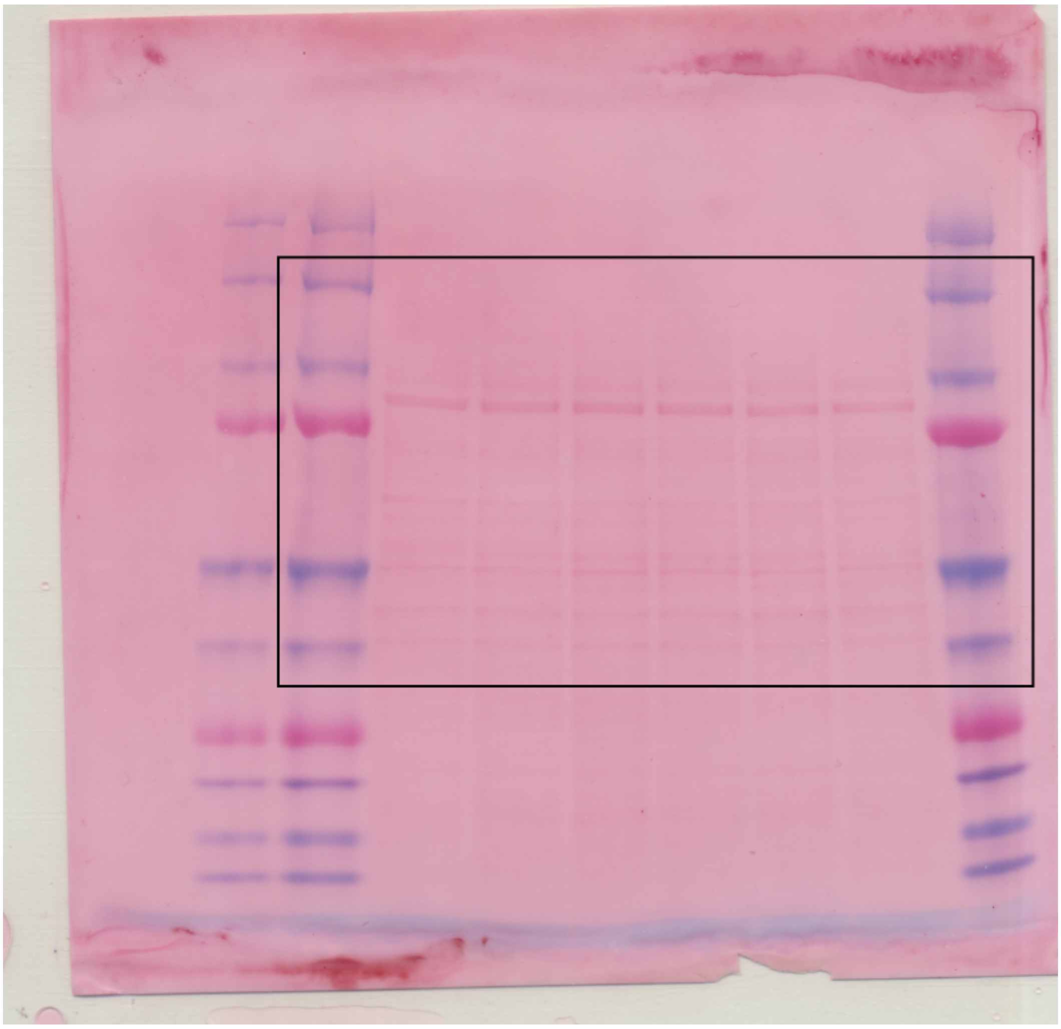

Figure S6A

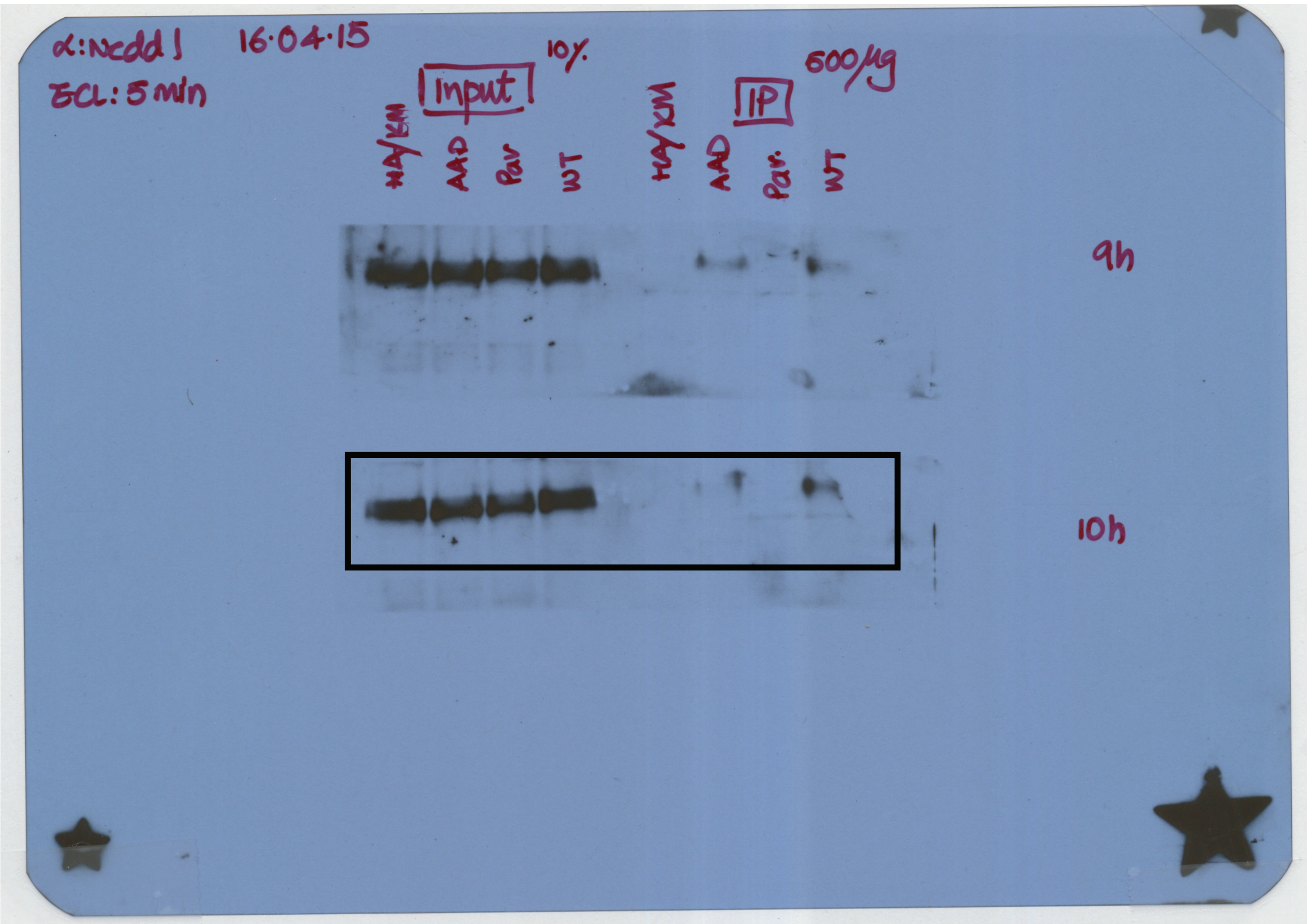
α:Nedd1
16·04·15
ECL: 5 min
10%
Input
600μg
IP
HA/KM
AAD
Par
WT
HA/KM
AAD
Par.
WT
9h
10h

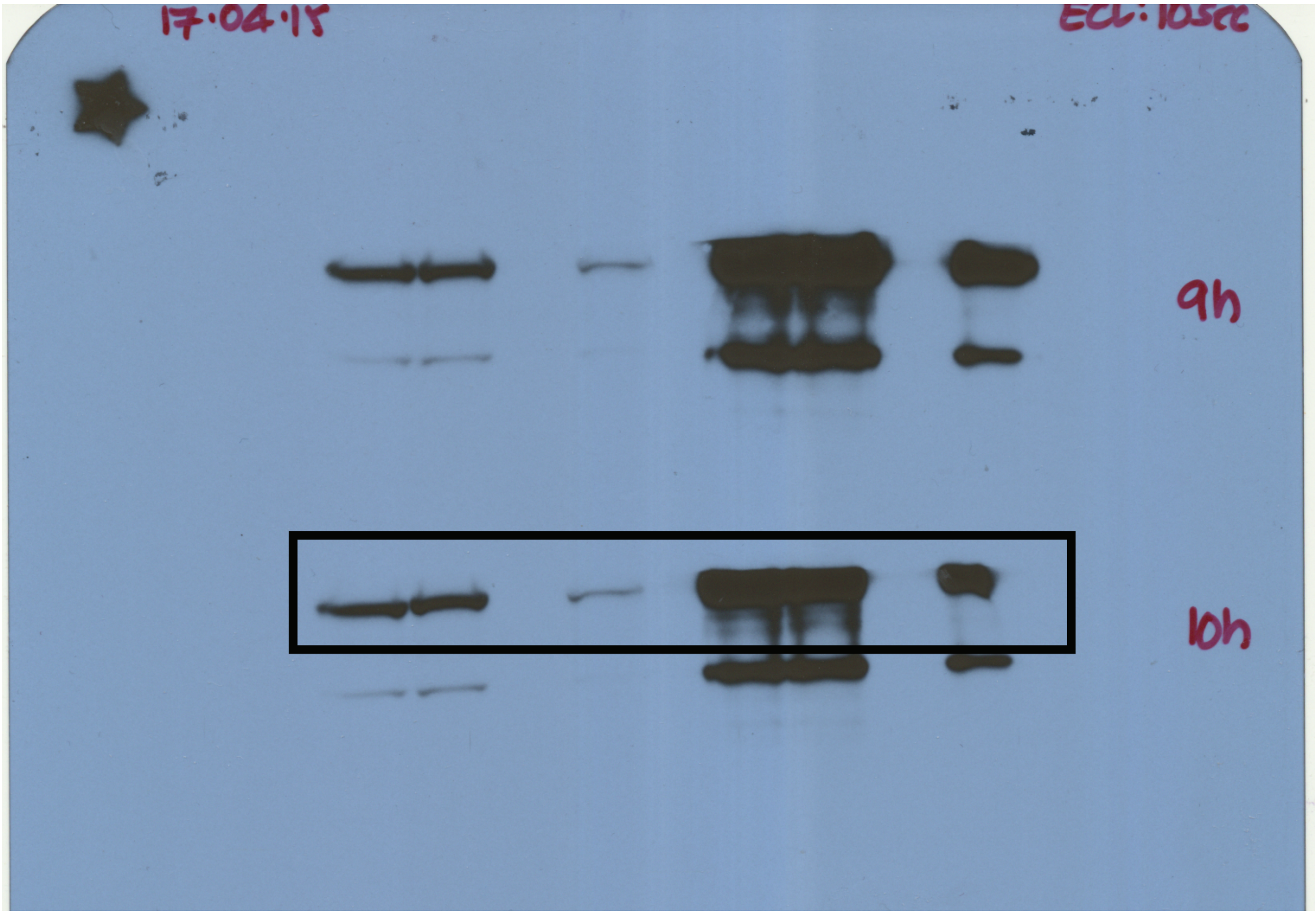
17·04·15
ECL: 10sec
9h
10h

Figure S6B

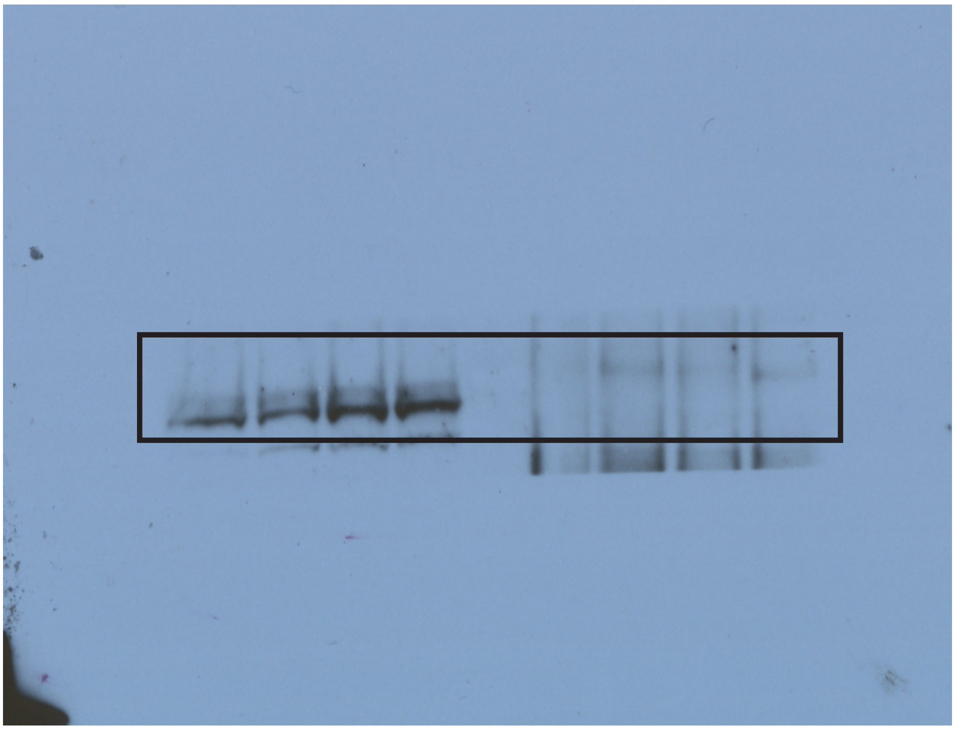

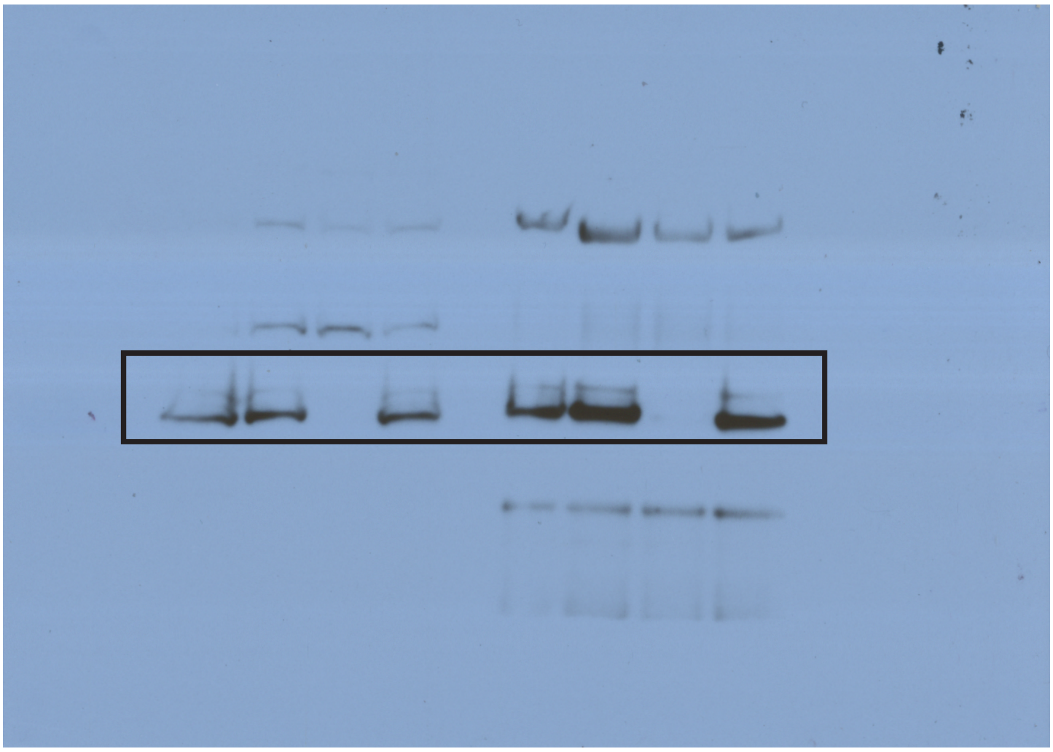

Figure S6C

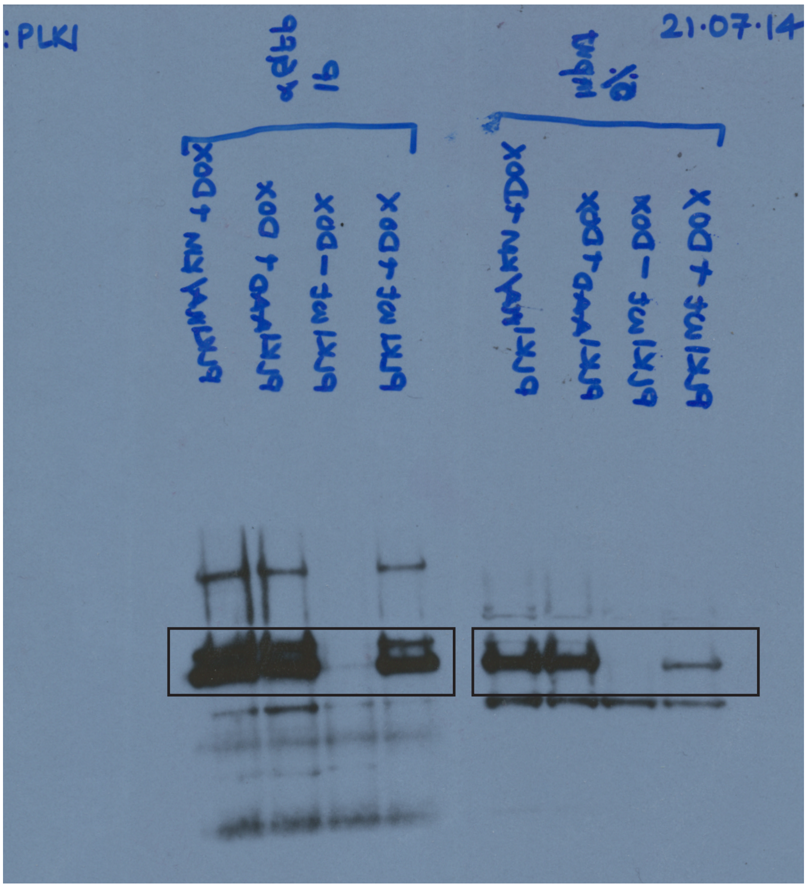

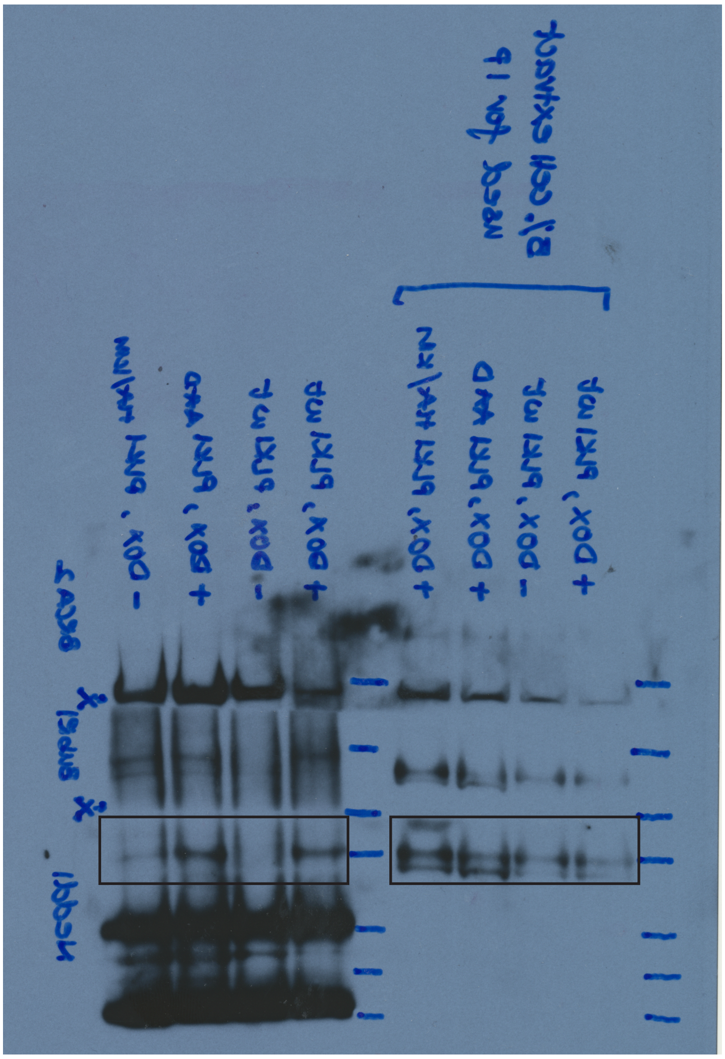

Figure S7

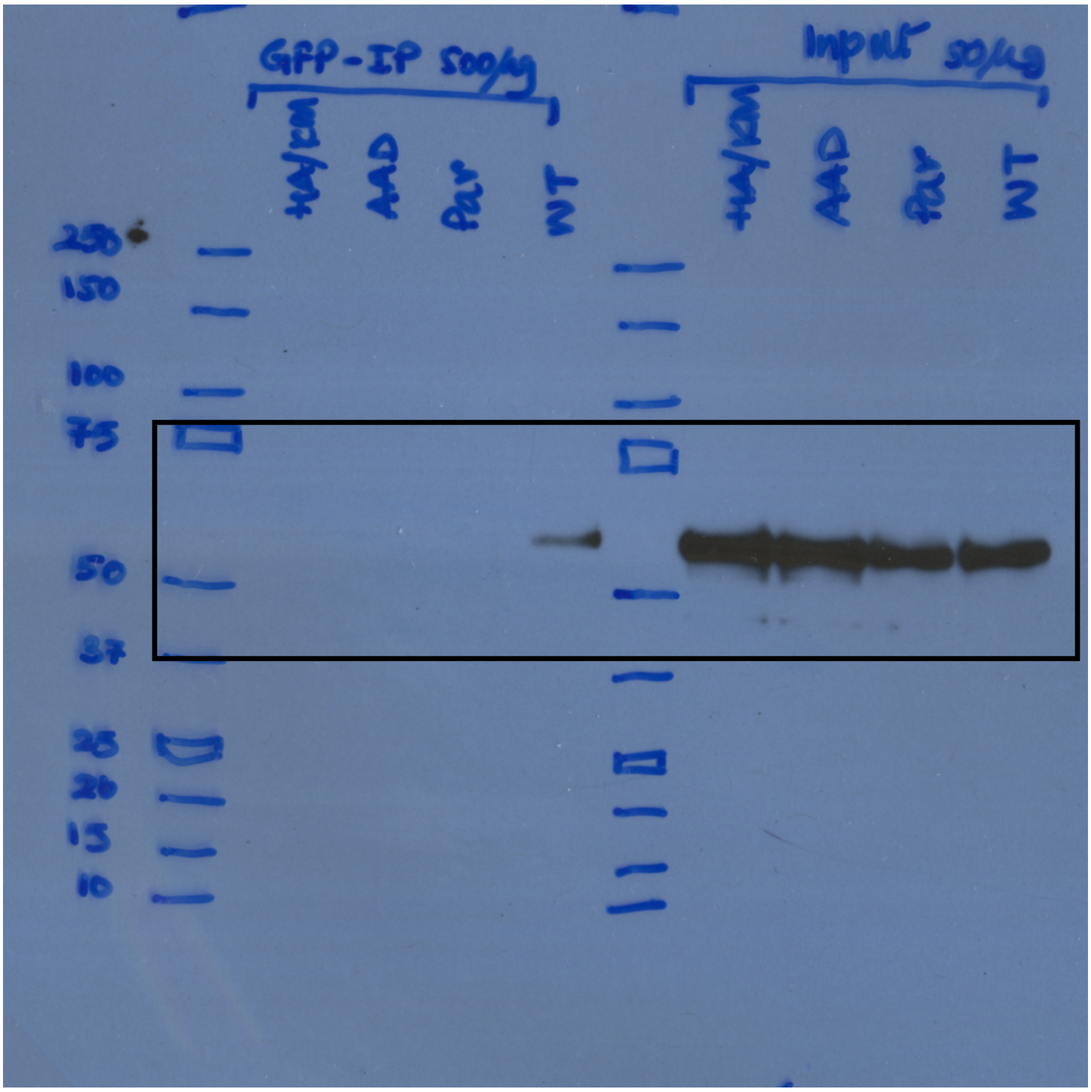
GFP-IP 500μg
Input 50μg
HA/KM
AAD
WT
HA/KM
AAD
WT
150
100
75
50
37
25
20
15
10

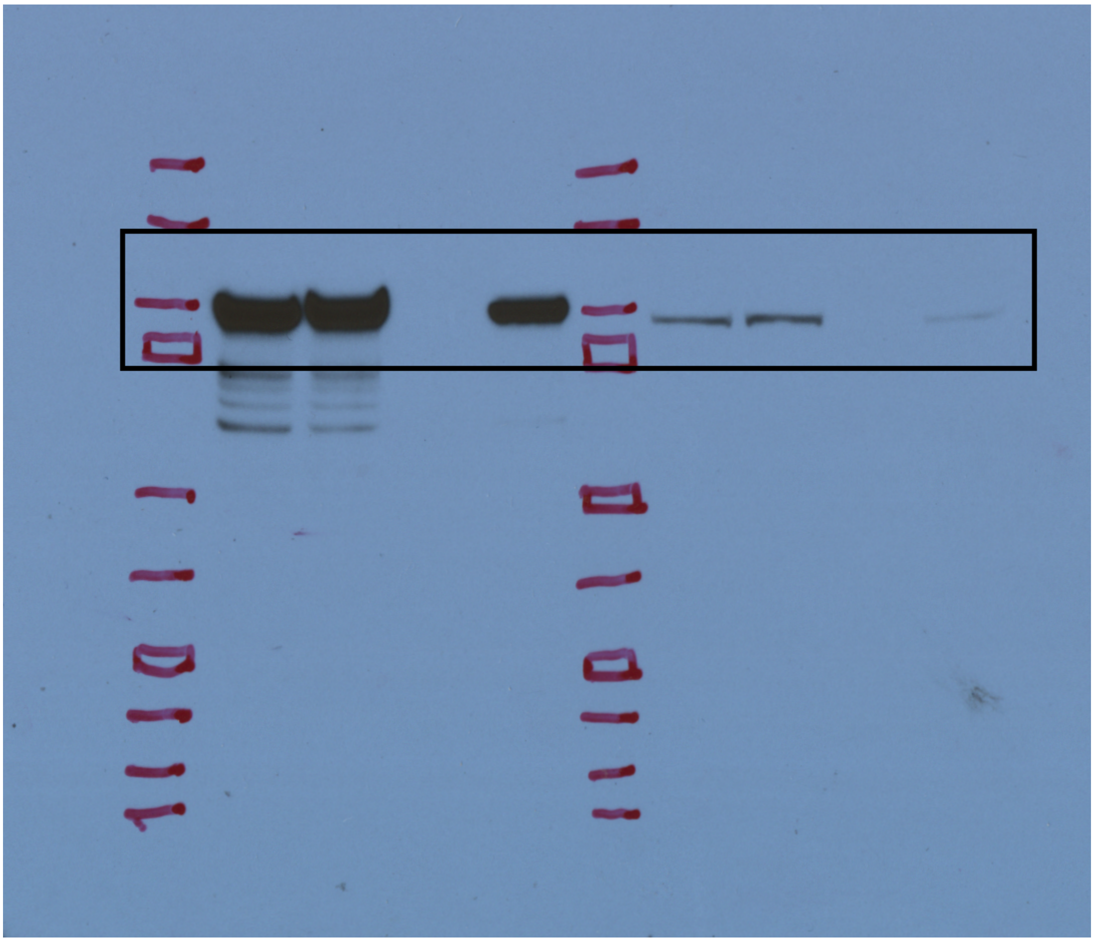

Supplement: Supplementary file 4 — Supplementary Information [file 41598_2019_50702_MOESM4_ESM.pdf]
